# PD1^hi^ CD200^hi^ CD4^+^ exhausted T cell increase immunotherapy resistance and tumour progression by promoting epithelial–mesenchymal transition in bladder cancer

**DOI:** 10.1002/ctm2.1303

**Published:** 2023-06-14

**Authors:** Chun Wu, Lianhui Duan, Hongmu Li, Xuefei Liu, Taonong Cai, Yang Yang, Yuting Yin, Wuguang Chang, Leqi Zhong, Lin Zhang, Yixin Cheng, Haide Qin, Zhesheng Wen, Huiyun Wang, Shijuan Mai

**Affiliations:** ^1^ State Key Laboratory of Oncology in South China Sun Yat‐sen University Cancer Center Guangzhou P. R. China; ^2^ School of Life Sciences Zhengzhou University Zhengzhou P. R. China; ^3^ Department of Thoracic Oncology Sun Yat‐sen University Cancer Center Guangzhou P. R. China; ^4^ School of Medicine Southern University of Science and Technology Shenzhen P. R. China; ^5^ Shenzhen Institute of Pediatrics Shenzhen Children's Hospital Shenzhen P. R. China; ^6^ Department of Urology Sun Yat‐sen University Cancer Center Guangzhou P. R. China; ^7^ School of Geography and Environmental Sciences Northwest Normal University Lanzhou P. R. China; ^8^ Department of Clinical Laboratory Sun Yat‐Sen University Cancer Center Guangzhou P. R. China; ^9^ Guangdong Provincial Key Laboratory of Malignant Tumor Epigenetics and Gene Regulation Sun Yat‐sen Memorial Hospital Sun Yat‐sen University Guangzhou P. R. China

**Keywords:** CD200, CD4 exhausted T cells, GAS6, N6‐methyladenosine

## Abstract

**Background:**

Bladder cancer (BLCA) is one of the most diagnosed cancers in humans worldwide. Recently, immunotherapy has become a main treatment option for BC. However, most BLCA patients do not respond to immune checkpoint inhibitors or relapse after immunotherapy. Therefore, it is very important to identify novel biomarkers for the prediction of immunotherapy response in B patients.

**Methods:**

Pancancer single‐cell RNA sequencing (scRNA‐seq) data were used to identify the clusters of CD4^+^ T cells in the tumour microenvironment (TME). The clinical significance of key CD4^+^ T‐cell clusters was evaluated based on the survival data of two independent immunotherapy bladder cancer (BLCA) cohorts. We also investigated the function of key clusters of CD4^+^ T cell in the TME of BC cells in vitro.

**Results:**

This study identified two novel exhausted CD4^+^ T‐cell subpopulations with the expression of PD1^hi^ CD200^hi^ or PD1^hi^ CD200^low^ in BC patients. Moreover, BLCA patients with a high level of PD1^hi^ CD200^hi^ CD4^+^ exhausted T cell showed immunotherapy resistance. Cell function analysis demonstrated that PD1^hi^ CD200^hi^ CD4^+^ exhausted T cell can promote epithelial–mesenchymal transition (EMT) and angiogenesis in BLCA cells. In addition, PD1^hi^ CD200^hi^ CD4^+^ exhausted T cells were shown to communicate with malignant BLCA cells through the GAS6–AXL axis. Finally, we also found that GAS6 expression is upregulated in B cells by METTL3‐mediated m6A modification.

**Conclusions:**

PD1^hi^ CD200^hi^ CD4^+^ exhausted T cell may serve as a novel biomarker for poor prognosis and immunotherapy resistance in B. Targeted inhibitors of PD1^hi^ CD200^hi^ CD4^+^ exhausted T cells may help improve the efficacy of immunotherapy.

## Graphical abstract

Graphical Abstract: We identified a novel population of CD4 tumour‐infiltrating lymphocytes (TILs) in BLCA, defined as the co‐expression of PD‐1 and CD200, which may help to explore the mechanisms of tumour progression and immunotherapy resistance. According to our study, patients with a high proportion of PD1hi CD200hi CD4^+^ exhausted T cells after immunotherapy suffered worse outcomes. We conclude that an immunotherapy/anti‐CD200 combination might offer a promising therapeutic option for bladder cancer patients with high proportions of PD1hi CD200hi CD4^+^ exhausted T cells.

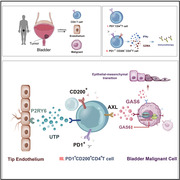

## 1 BACKGROUND

An estimated 81 180 new cases of urinary bladder cancer (61 700 men and 19 480 women) will be diagnosed in the United States in 2022, with approximately 17 100 deaths (12 120 men and 4 980 women) occurring during this same period.^1^ Bladder cancer has become an increasingly prominent public health issue due to its high metastatic propensity and increased immunotolerance.^2^, ^3^ In the treatment of bladder cancer, immune checkpoint inhibitors (ICIs) targeting CTLA‐4 and PD‐1 have proven to be an effective strategy.^4^, ^5^, ^6^ At present, the mechanism of antitumour immunity induced by immunotherapy mainly focuses on CD8^+^ exhausted T cells.^7^ However, ICIs monotherapy has only an efficacy of only 20% in bladder cancer patients.^6^, ^8^, ^9^ An increasing number of studies have shown that CD4^+^ T cells are susceptible to exhaustion and may contribute to the reactivation of the antitumour immune response after ICI treatment in multiple cancer types, including bladder cancer.^10^, ^11^ Therefore, it is necessary to explore the role of CD4^+^ exhausted T cells in bladder cancer.

Upon antigen‐driven activation, naive CD4^+^ T cells expand and differentiate into a broad spectrum of functional subsets, including T‐helper 1 (Th1), T‐helper 2 (Th2), T‐helper 9 (Th9), T‐helper 17 (Th17), regulatory T cells (Tregs) and follicular T helper (TFH) cells.^12^, ^13^ Activation of different T helper cell subtypes may exert contradictory effects on tumour immune response. Th1 cells can not only assist CD8^+^ T cells but also promote tumour vessel normalization by secreting IFN‐γ, to inhibit cancer progression.^14^, ^15^ In contrast, regulatory CD4^+^ T cells (Tregs) can promote cancer development and progression by inhibiting the immune response against tumours. In addition, Tregs participate in the epithelial–mesenchymal transition (EMT) of cancer cells and angiogenesis by secreting growth factors^15^, ^16^ and support the growth of stromal cells (such as fibroblasts and endothelial cells).^17^, ^18^ Moreover, CD4^+^ T cells seem to be highly plastic under the influence of specific cytokines, allowing them to switch between subsets, thereby extending their functional range.^19^

In this study, we identified PD1^hi^ CD200^hi^ CD4^+^ exhausted T cells in bladder cancer, which are closely associated with poor prognosis and immunotherapy resistance. Moreover, PD1^hi^ CD200^hi^ CD4^+^ exhausted T cells can promote EMT and angiogenesis in bladder cancer cells in vitro. Finally, we observed that malignant cells can recruit PD1^hi^ CD200^hi^ CD4^+^ exhausted T cells to induce EMT in bladder cancer cells by releasing m6A‐mediated GAS6.

## 2 MATERIALS AND METHODS

### 2.1 Single‐cell transcriptome sequencing collection and data preprocessing

#### 2.1.1 Pan‐CD4 single‐cell transcriptome sequencing

Single‐cell transcriptome sequencing (scRNA‐seq) data have been downloaded or requested from the authors of published studies. The details of each dataset are shown in Tables S1 and S2. For each sample, we used the Cell Ranger Single‐Cell toolkit to align reads for each sample based on the human reference genome GRCh38 (https://cf.10xgenomics.com/supp/cell‐exp/refdata‐gex‐GRCh38‐2020‐A.tar.gz).

The Seurat (v3.1.3) R toolkit was used to analyse scRNA‐seq data for each cancer type.^20^ First, cells with more than 20% mitochondrial RNA were removed, as well as cells with UMI less than 200 or UMI greater than 6000. ‘DoubletFinder’^21^ was used to predict and remove doublets. For each patient, we considered the rate of doublet cells to be filtered to be 4%, using five principal components and a default value of 20% for pN.

Then, the scRNA‐seq data for each cancer type were normalized for each sample using the ‘NormalizeData’ function in Seurat, and only the highly variable genes were retained using the ‘FindVariableFeatures’ function in Seurat. Next, the ‘Runharmony’ function in the harmony package was used to integrate the gene expression matrix of all samples, which adjusts for batch effects between samples.^22^ The ‘RunPCA’ function was used to perform principal component analysis (PCA), and the ‘FindNeighbors’ function is used to construct a *K*‐nearest neighbour graph. The most representative principal components (PCs) selected based on PCA were used for cluster analysis with the ‘FindCluster’ function to identify different cell types. Subclusters with high expression of CD3E, CD3G and CD3D were annotated as T cells. Separation of CD4^+^ T lymphocyte subclusters from T lymphocytes based on CD4 expression. We combined the expression matrix of CD4^+^ T lymphocytes of all cancer types and used the above clustering approach to obtain a pancancer CD4^+^ T lymphocyte map. In this step, we also used the Runharmony function to extract batch differences for each patient in each dataset.

Differentially expressed genes in each subcluster were identified based on the ‘wilcoxauc’ function in the presto package. The log‐transformed fold change (log2FC), which was used to determine the magnitude of the difference, was taken into account in our single‐cell RNA sequencing analysis. We considered differentially expressed genes with log_2_FC > .3 and *p‐*values <.05 to be potentially biologically significant.

#### 2.1.2 Endothelial and epithelial cell single‐cell transcriptome sequencing of BLCA datasets

We used the same approach as pan‐CD4 scRNA‐seq to analyse scRNA‐seq data from two BLCA datasets and identified eight major clusters and annotated them according to the expression of typical gene markers, including endothelial, epithelial, fibroblast, myeloid, mast, plasma, B and T cells. CD4, CD8A and CD8B gene expressions were used to define CD4^+^ and CD8^+^ T cells. Endothelial cell and epithelial cell subclusters were named using the first marker gene.

### 2.2 Tissue preference statistic

In this paper, we use Ro/e values to estimate the tissue preference of each subcluster, with Ro/e denoting the ratio of the number of cells observed in a cluster to the expected number of cells. The expected number of cells per cell cluster in each tissue was obtained from the *χ* ^2^ test. Ro/e > 1 for a subcluster in a given tissue indicates that the cluster is enriched in that tissue.

### 2.3 Bulk RNA sequencing data collection and processing

Bulk RNA sequencing transcriptome data of patients with BLCA and the corresponding clinical data and mutation profiles were downloaded from The Cancer Genome Atlas (TCGA) database (https://portal.gdc.cancer.gov/, accessed on 27 October 2022). Fragments per kilobase million values were converted to transcripts per kilobase million values, and the Ensembl gene IDs of the RNA‐seq data were converted to gene symbols concerning the annotation file. By using the median value of PDCD1 and CD200 gene expression of each patient, we first divided the patients into two groups, the PDCD1^hi^ group and the PDCD1^low^ group, and then divided the PDCD1^hi^ group into two groups according to the expression of CD200.

We included two immunotherapeutic BLCA cohorts and obtained the relative transcriptomic and clinical data from the online Supporting Information section appended to the published studies. We downloaded RNA sequencing data from the immunotherapy BLCA cohort (IMvigor210) from the Mvigor210CoreBiologies package.^9^ Another immunotherapy BLCA cohort (GSE176307) was downloaded from the Gene Expression Omnibus (GEO) database (http://www.ncbi.nlm.nih.gov/geo/, accessed on 27 October 2022) and included for analysis.^23^ In addition, we included five additional immunotherapeutic cohorts with different types of cancer and obtained relevant transcriptomic and clinical data from online Supporting Information section from published studies. RNA sequencing data from an immunotherapy melanoma cohort (SKCM, GSE91061)^24^ and non‐small cell lung cancer cohort (NSCLC, GSE135222)^25^ were downloaded from GEO. The raw data were downloaded as microarray data. Another immunotherapy melanoma cohort (SKCM, phs00052) was included in the analysis and downloaded from the Database of Genotypes and Phenotypes (dbGaP, https://dbgap.ncbi.nlm.nih.gov/, accessed on 20 November 2022). An immunotherapy cohort of stomach adenocarcinoma (STAD, PRJEB25780) was included for analysis and downloaded from the European Nucleotide Archive dataset (ENA, https://www.ebi.ac.uk/ena/browser/home, accessed on 20 November 2022). Finally, the data from an immunotherapy renal cell carcinoma cohort (RCC, Braun_2020) were included and are shown in Table S5.

### 2.4 Pathway enrichment analysis of bulk RNA‐seq and scRNA‐seq

The identification of differentially expressed genes between the CD4_ex1 and CD4_ex2 subclusters was carried out using the Limma R package.^26^ To explore the phenotype‐specific signalling pathways of the tumour microenvironment (TME) in the CD4_ex1 subcluster, the gene set enrichment analysis (GSEA) approach was used with an adjusted *p* < .05 using the ‘clusterProfiler’ R package.^27^ Next, we also used the Limma R package to identify differentially expressed genes between the PDCD1^hi^ CD200^hi^ group and the other two groups. We also used the ‘clusterProfiler’ R package to probe the hallmark signalling pathway in the PDCD1^hi^ CD200^hi^ group with an adjusted *p* < .05.

### 2.5 Immunological characteristics of the TME in BLCA cohort

The proportions of immune cell types evaluated for immune cell infiltration in each sample (with immune infiltration scores) were computed using the CIBERSORT (https://cibersort.stanford.edu/) and the Xcell algorithm.^28^

### 2.6 Cell–cell communication analysis

Cell–cell communication between different cell subclusters was analysed with CellChat (Version 1.1.3, R package) following the standard protocol.^29^ We used the CellChat package to quantitatively characterize and comparatively infer the probability of cell–cell communication between the CD4_ex2 subcluster and all subclusters of epithelial cells, as well as the ligands and receptors for intercellular communication.

### 2.7 Cell developmental trajectory analysis

RNA velocity analysis was conducted by using velocyto and scVelo.^30^ First, we used the 10× velocyto pipeline to count spliced and unspliced reads for each sample from cellranger‐generated BAM files. To predict the root and terminal states of the underlying Markov process, the respective scVelo functions were applied. We also used the Python package PAGA to verify the pseudotime between each epithelial cluster.^31^

Pseudotime trajectory analysis was constructed based on Monocle2^32^ (version 2.18.0, R package) following the tutorial to order the three epithelial cell subclusters (Epi_CXCL1, Epi_OLFM4 and Epi_COL1A2) in BLCA data. To avoid omitting some important genes, we selected all genes for completely unsupervised trajectory analysis. For the differentiation trajectory analysis, we selected the top 50 pseudotime‐related differentially expressed genes for these 3 clusters for further analysis.

### 2.8 Metabolic cell–cell communication

We used MEBOCOST which is a Python‐based computational tool to infer metabolite, mediated cell–cell communication events. The cut‐off parameter was set to .25. Other parameters were set as default.

### 2.9 Patients and tissue samples

Peripheral blood and tumour samples were obtained from the Sun Yat‐sen University Cancer Center, Sun Yat‐sen University, Guangzhou, China. The patients included in this study were as follows: 18 BLCA patients, 10 non‐small cell lung carcinoma (NSCLC) patients and 8 esophageal squamous cell cancer (ESCC) patients. All patients were pathologically diagnosed and collected with informed consent. This study was performed with the approval of the Ethics Committee of Sun Yat‐sen University Cancer Center (GZR2018‐053 and GZR2018‐120).

### 2.10 Cell lines

The human microvasculature endothelial cell line HMEC1 was purchased from Biospecies, Ltd. with cell authentication via the STR multiamplification method. Human bladder cancer cells UMUC‐3 and T24 were obtained from Sun Yat‐sen University Cancer Center. UMUC‐3 and T24 cell lines were maintained in RPMI‐1640 medium (Gibco, Canada) supplemented with 10% foetal bovine serum (Sigma, USA). HMEC‐1 cell line specialty medium (Biospecies, Guangzhou, China). All cell lines were cultured in a humidified atmosphere of 5% CO2 at 37°C. Indirect coculture assays were performed using .4 μm cell culture inserts (Corning, NY, USA).

### 2.11 Primary human T‐cell isolation and culture

Human CD200^hi^ CD4^+^ T cells and CD200^low^ CD4^+^ T cells were obtained from PBMCs with a negative CD4 magnetic selection Kit (StemCell Technologies, Vancouver, CA, USA) and a human PE Position Selection Kit (StemCell Technologies, Vancouver, CA, USA). To increase the expression level of PD1, the T cells were cultured in the presence of 2 μg/mL anti‐CD3 antibodies (PeproTech, Rocky Hill, NJ, USA), 2 μg/mL anti‐CD28 antibodies (PeproTech) and 10 ng/mL IL2 (PeproTech). Forty‐eight hours later, the purity (>98%) of PD1^hi^ CD200^hi^ CD4^−^ T cells and PD1^hi^ CD200^low^ CD4^−^ T cells were confirmed by using flow cytometry.

### 2.12 Flow cytometry

Human PD1^hi^ CD200^hi^ CD4^+^ exhausted T cells and PD1^hi^ CD200^low^ CD4^+^ exhausted T cells were stimulated with Leukocyte Activation Cocktail (BD Biosciences, San Jose, CA, USA) for 4 h. Cells were harvested by using staining buffers (BD Biosciences, San Jose, CA, USA). To reduce background staining, the cells were cultured with 2 μg/mL Fc receptor blocker (Absin, China) for 15 min before the addition of antibodies. Cell‐surface staining was performed with anti‐CD4‐APC (BD Biosciences, San Jose, CA, USA), anti‐PD1‐BV421 (BD Biosciences, San Jose, CA, USA) and anti‐CD200‐PE (BioLegend, USA). Cells were incubated at 4°C for 30 min and then washed in fluorescence‐activated cell sorting (FACS) buffer. To facilitate intracellular staining, cells were fixed and permeabilized with a fixation/permeabilization solution kit (BD Biosciences, San Jose, CA, USA). After fixation and permeabilization, cells were stained with anti‐IFNG‐PerCP‐Cy5.5 (BD Biosciences, San Jose, CA, USA). Cells were incubated at 4°C for 30 min and then washed and resuspended in FACS buffer. The results were analysed using the FlowJo 10.7 software program.

### 2.13 Cell invasion assays

For the in vitro invasion assays, the upper chambers of Transwells plates (Corning, NY, USA) were coated with 100 μL diluted Matrigel (Corning, NY, USA). Before the invasion assays, T24 and UMUC3 cells (1 × 10^5^) were cocultured with PD1^hi^ CD200^hi^ CD4^+^ exhausted T cells/PD1^hi^ CD200^low^ CD4^+^ exhausted T cells (1 × 10^6^) for 48 h in Transwell plates. Two hundred microliters of T24 and UMUC3 cells (1 × 10^5^) in serum‐free media and 1% serum‐containing media were plated in the upper and lower chambers, respectively. After 48 h of incubation, the cells in the upper chamber were removed. Invasive cells located on the lower side of the chamber were fixed with 100% methanol for 10 min and stained with crystal violet for 30 min at room temperature. Four random fields per well were observed, and cells were counted under a microscope. Experiments were performed in triplicate.

### 2.14 Matrigel tube formation assay

For the in vitro tube formation assays, 48‐well culture plates were coated with 150 μL of Matrigel (Corning, NY, USA) per well and then allowed to polymerize for 30 min at 37°C. Before the tube formation assays, HMEC1 cells (2 × 10^5^) were cocultured with PD1^hi^ CD200^hi^ CD4^+^ exhausted T cells/PD1^hi^ CD200^low^ CD4 exhausted T cells (1 × 10^6^) for 48 h in Transwell plates. After coculture, 200 μL HMEC1 cells (5 × 10^4^) were seeded on polymerized Matrigel. After incubation at 37°C for 4 h, each culture was photographed by using a confocal microscope (Leica, Wetzlar, Germany) to capture four random fields per well.

### 2.15 RNA interference

Small interfering RNA (siRNA) duplexes targeting METTL3 (siRNA1400 and siRNA1640) and the negative control (NC) siRNA were designed and synthesized by GenePharma (Shanghai, China). Cell transfection was achieved using Lipofectamine 3000 (Invitrogen, CA, USA) according to the manufacturer's instructions. Briefly, a 100 nM siRNA duplex was incubated with cell lines for 48 h for each transfection, after which the following experiments and assays were performed.

### 2.16 Western blotting

Cells were lysed in RIPA lysis buffer (Biyuntian, Hangzhou, China) containing 1% protease and phosphatase inhibitors (Thermo Fisher Scientific, MA, USA). Protein concentration was measured using a BCA Protein Assay Kit (Biyuntian, Hangzhou, China). Samples (30 μg) were separated by electrophoresis on 8%–15% SDS‒PAGE gels and then transferred onto PVDF membranes (.45 μm; Merck‐Millipore, Darmstadt, Germany) using a wet transfer system (Bio‐Rad, Hercules, CA, USA). After blocking with blocking solution (Biyuntian, Hangzhou, China) for 15 min at room temperature, the membranes were incubated overnight with primary antibodies at 4°C, followed by secondary antibodies for another hour. Blots were detected by the enhanced chemiluminescence system (Millipore, MA, USA).

### 2.17 Quantitative real‐time PCR

The mRNA expression of key genes in bladder cancer was determined using quantitative real‐time PCR (qRT‐PCR). Total RNA was extracted using TRIzol reagent (Sigma‐Aldrich, St Louis, MO, USA). cDNA was synthesized from total RNA using a cDNA reverse transcription kit (Thermo Fisher Scientific, MA, USA). qRT‐PCR was performed using the SYBR Green PCR kit (Thermo Fisher Scientific, MA, USA). The housekeeping gene GAPDH was used as an endogenous control. Primer information: GAS6: 5′‐GGTAGCTGAGTTTGACTTCCG‐3′ (forward) and 5′‐GACAGCATCCCTGTTGACCTT‐3′ (reverse); GAPDH: 5′‐GGAGCGAGATCCCTCCAAAAT‐3′ (forward) and 5′‐GGCTGTTGTCATACTTCTCATGG‐3′ (reverse). The cycle threshold (*Ct*) of each gene in samples was recorded. Relative quantification was calculated as 2^−Δ^ *^Ct^* (Δ*Ct* values  =  target gene mean *Ct* value – control gene mean *Ct* value).

### 2.18 Methylated RIP analysis

TRIzol was used to extract total RNA from bladder cancer cells transfected with 100 nM siMETTL3 or control siRNA. Then, the Magna MeRIP m6A Kit (RiboBio, Guangzhou, China) was used for methylated RNA immunoprecipitation (RIP) according to the manufacturer's protocol. After that, an RNA purification kit (Zymo Research Corp., Irvine, CA, USA) was used to extract the enriched RNA. The enrichment of m6A‐containing RNA relative to the negative (IgG) sample was analysed by qRT‐PCR with the primers GAS6: 5′‐GGTAGCTGAGTTTGACTTCCG‐3′ (forward) and 5′‐GACAGCATCCCTGTTGACCTT‐3′ (reverse).

### 2.19 RNA immunoprecipitation analysis

RIP was performed with antibodies specific for METTL3 by using the Magna RIP RNA‐Binding Protein Immunoprecipitation Kit (Merck‐Millipore, Darmstadt, Germany) according to the manufacturer's protocol. In brief, 4 × 10^7 bladder cancer cells were harvested and lysed in RIP lysis buffer. After centrifugation at 4°C, the supernatant was incubated with METTL3 antibodies and NC IgG at room temperature. Then, the bead‐antibody complex was washed and incubated with Proteinase K buffer. The input and immunoprecipitated RNAs were isolated by TRIzol reagent and reverse transcribed into cDNA using the RevertAid First Strand cDNA Synthesis Kit (Thermo Fisher Scientific, MA, USA). The fold enrichment relative to the negative (IgG) sample was measured by qRT‐PCR with the primers GAS6: 5′‐GGTAGCTGAGTTTGACTTCCG‐3′ (forward) and 5′‐GACAGCATCCCTGTTGACCTT‐3′ (reverse).

### 2.20 Immunofluorescence assay and spatial analysis

Multiplex immunofluorescence staining was performed using the PANO 7‐plex IHC kit (Panovue, Beijing, China). In detail, the slides were incubated for 2 h at 65°C, deparaffinized with xylene and ethanol diluted in a graduated series (100%, 95%, 70%, 50%) and then fixed in 10% neutral buffered formalin for 30 min. After that, microwave antigen retrieval was performed using EDTA antigen repair buffer (pH = 9.0, ZSGB‐Bio, Beijing, China), followed by blocking. Following those procedures, horseradish peroxidase (HRP)‐conjugated secondary antibodies were incubated, and tyramide signal amplification was carried out after sequentially applying different primary antibodies, including PD1 (CST, Boston, MA, USA), CD4 (CST, Boston, MA, USA), CD200 (CST, Boston, MA, USA), CD31 (CST, Boston, MA, USA), GSA6 (CST, Boston, MA, USA) and IFNG (Novus, USA). The incubation temperature, time and dilution are listed in Table S14. Subsequently, sections were incubated using biotinylated rabbit polyclonal anti‐rabbit and rabbit anti‐mouse antibodies as secondary antibodies followed by HRP‐conjugated streptavidin treatment according to the manufacturer's instructions (Panovue, Peking, China). Biotinylated secondary antibodies were used for streptavidin‐linked alkaline phosphatase–dependent chromogen reactions for streptavidin‐linked fluorophores for IF (excitation wavelengths of 480, 520, 570, 690 or 780 nm, Table S14). Multispectral images were acquired using the Vectra Polaris Automated Quantitative Pathology Imaging System (Akoya Biosciences, State of Delaware, USA).

To calculate the spatial relationship between different cells, inferences are made about the interactions between cells. Proximity analysis in HALO software (Indica Labs, Corrales, NM, USA) was performed to assess the microenvironment around PD1^hi^ CD200^hi^ CD4^+^ exhausted T cells in bladder cancer. The PD1^hi^ CD200^hi^ CD4^+^ exhausted T cells were registered and plotted together as a single figure. The spatial analysis tool was used to calculate the average distance between PD1hi CD200hi CD4 exhausted T cells and CD31^+^, IFNG^+^ and GAS6^+^ cells. The 50 μm cut‐off was selected based on analysis from previous studies.^33^ Spatial data for all analyses were exported for statistical analysis.

### 2.21 The construction of the mouse model

The subcutaneous syngeneic BLCA mouse models were generated by injecting 5 × 105 MB49 cells in 100 μL PBS into 5–6‐week‐old male C57BL/6J mice (*n* = 15). Tumour sizes were measured using callipers every day in an unblinded manner, and tumour volumes were calculated (*V* = .5 × *L* × *W*2). Once tumours reached 50 mm^3^, animals were randomly assigned to receive anti‐PD1 (anti‐murine PD‐1 mAb clone, BioXCell BE0273) or PBS. The MB49 model was used as validation, and all animals were sacrificed 8 days after drug administration. Tumours were collected and processed for haematoxylin–eosin staining and mIHC assays. All animal studies were approved by the laboratory animal ethics committee of Sun Yat‐sen University Cancer Center.

### 2.22 Statistical analysis

Using the Kaplan–Meier method to estimate overall survival (OS), FPI or DSS, the Kaplan–Meier curves were compared using the log‐rank test. A two‐sided *p*‐value of less than .05 was regarded as significant. Moreover, all of the sample sizes were large enough to enable proper statistical analysis. Spearman correlation analysis was applied in all of the correlation analyses. GraphPad Prism (https://www.graphpad.com/, accessed on 25 November 2022) was also used to perform statistical analyses. *p*‐Values less than .05 were deemed to be statistically significant. All of the *t* tests were two‐sided *t* tests (paired or unpaired, depending on the experiments).

## 3 RESULTS

### 3.1 Two subclusters of CD4^+^ exhausted T cells exist in the tumour microenvironment

**FIGURE 1:**
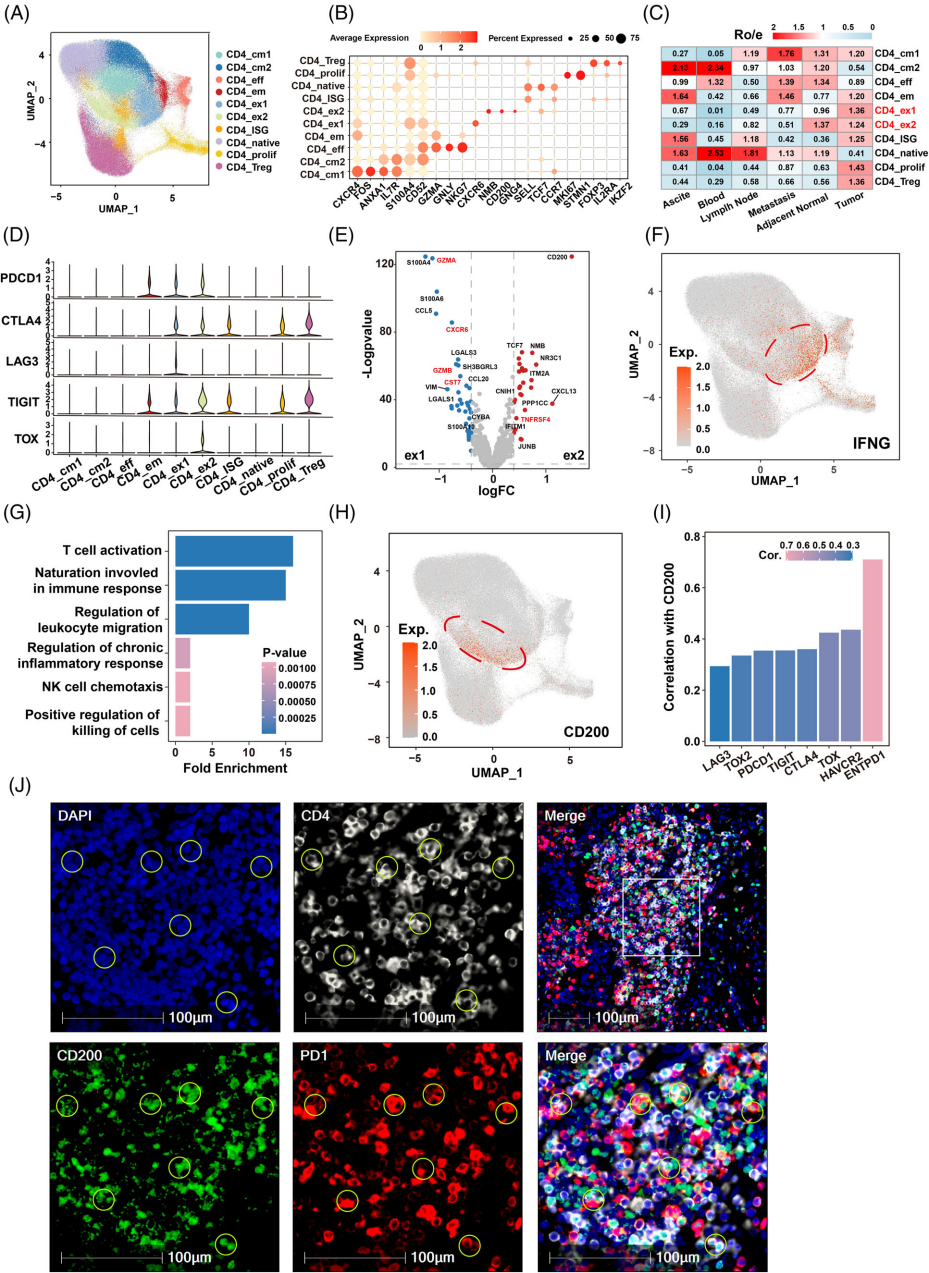
Two subclusters of CD4^+^ exhausted T cells were defined in the tumour microenvironment using the pancancer CD4^+^ T cell atlas: (A) the UMAP plot of the subclusters of CD4^+^ T cells integrated from nine tumour types. Each dot indicated a single cell. Colour‐coded for the cell type; (B) the dot plot showing the particular genes for each subcluster of CD4^+^ T cells; (C) tissue prevalence estimated by Ro/e score in pan‐CD4^+^ T cells; (D) the violin plot showing exhausted‐related genes expression in each cluster; (E) volcano map showing differentially expressed genes between CD4_ex2 and CD4_ex1 subclusters (ex2: logFC > .3 and log *p*‐value >2; ex1: logFC < −.3 and log *p*‐value >2); (F) the UMAP plot showing the IFNG expression in pan‐CD4^+^ T cells; (G) the barplot showing the pathway enrichment of CD4_ex1 subcluster; (H) the UMAP plot showing the CD200 expression in pan‐CD4^+^ T cells; (I) Barplot** showing the correlation between T cell exhausted‐related genes and CD200 in The Cancer Genome Atlas (TCGA)‐BLCA; (J) multiplex immunofluorescence staining was performed for DAPI (blue), CD4 (white), PD1 (red) and CD200 (green); each stain was shown separately and merged. The circles represent the PD1^hi^ CD200^hi^ CD4^+^ exhausted T cells. The field of view in the square is the reduced field of view.

In this study, we created an atlas of CD4^+^ T‐cell transcriptomes using 161 660 CD4^+^ T cells derived from 6 immune‐relevant sites: tumour, adjacent normal tissues, distant tumour metastasis, lymph node, peripheral blood and ascites, using previously published scRNA‐seq datasets from patients with 8 types of cancer, including basal cell carcinoma (BCC), bladder cancer (BLCA), clear cell renal carcinoma (ccRCC）, colorectal cancer (CRC), esophageal squamous cell carcinoma (ESCC), head‐and‐neck squamous cell carcinoma (HNSC), nasopharyngeal carcinoma (NPC) and lung cancer (Figure S1A,B; Tables S1 and S2). The unsupervised clustering analysis identified 10 distinct CD4^+^ T‐cell clusters and we identified the 10 clusters according to CD4^+^ T‐cell subcluster signature genes. The 10 clusters included 2 clusters of central memory CD4^+^ T cells, effector CD4^+^ T cells, effector memory CD4^+^ T cells, CD4^+^ exhausted T cells 1 (CD4 Tex1), CD4^+^ exhausted T cells 2 (CD4 Tex2), interferon‐stimulated gene‐related CD4^+^ T cells, naive CD4 T cells, proliferating CD4^+^ T cells and regulatory CD4^+^ T cells (Figure 1A,B; Figure S1C; Table S1). Next, the quantified tissue enrichment analysis indicated that the CD4 Tex1 and CD4 Tex2 clusters were mainly enriched in all eight types of cancer (Figure 1C; Figure S1D,E). Furthermore, the investigation of the expression of exhaustion marker genes revealed that common exhaustion markers (PDCD1, CTLA4, LAG3, TIGIT and TOX) were specifically expressed in the CD4 Tex1 and CD4 Tex2 subclusters, confirming their exhausted states (Figure 1D; Figure S1F; Table S3).

Differential gene expression analysis revealed that no immuno‐effecting properties were associated with the CD4 Tex2 cluster. Moreover, CD4 Tex2 cells have significantly higher expression of TNFRSF4 and TCF7 than that of CD4 Tex1 cells, indicating their Treg‐like activity and a certain naive state, respectively.^34^, ^35^, ^36^ In contrast, GZMA and CST7 were specifically expressed in the CD4 Tex1 cluster, suggesting that the subcluster has an immunological effect to a certain extent (Figure 1E; Table S4). Although the CD4 Tex1 cluster was considered to contain cells in an exhausted state, it still secreted certain factors that promote its antitumour function, including IFNG (Figure 1F). GO biological pathway enrichment analysis also revealed that the CD4 Tex1 cluster was predominantly involved in T cell activation (Figure 1G). Additionally, based on the differential gene expression profiles, CD4_Tex1‐expressing genes were related to those of Th17 cells, including IL17A, IL17F, RORC, RORA and IL22,^37^, ^38^ whereas CD4_Tex2‐expressing genes exhibited features similar to those of Tfh cells, including BCL6, CXCR5, ICA1, IL6ST and MAGEH1^39^, ^40^, ^41^ (Figure S1G).

Interestingly, the CD4 Tex2 cluster is characterized by high levels of CD200 expression. CD200 was specifically highly expressed in the CD4 Tex2 cluster compared with CD4 Tex1 and all the other subclusters of CD4 T cells (Figure 1H; Figure S1H). Therefore, the CD4 Tex1 cluster was defined as PD1^hi^ CD200^low^ CD4 exhausted T cells and the CD4 Tex2 cluster was defined as PD1^hi^ CD200^hi^ CD4 exhausted T cells. In addition, we discovered that CD200 expression was significantly and positively correlated with the exhaustion‐associated molecules ENTPD1, HAVCR2 and TOX in TCGA (Figure 1I). Finally, multiplex immunofluorescence was performed to determine the infiltration of PD1^hi^ CD200^hi^ CD4 exhausted T cells in bladder cancer tissue, which showed that PD1^hi^ CD200^hi^ CD4 exhausted T cells were commonly present in the tumour‐infiltrating CD4^+^ T cells in the bladder cancer tissues (Figure 1J). In addition, PD1^hi^ CD200^low^ CD4 and PD1^hi^ CD200^hi^ CD4 exhausted T‐cell subsets were also isolated from the blood of 18 bladder cancer patients, 10 non‐small cell lung carcinoma patients and 8 esophageal squamous cell carcinoma patients by magnetic sorting (Figure S1I).

In summary, we identified two novel subclusters of CD4^+^ exhaustion T cells with substantially different properties. The CD4^+^ Tex1 cluster with a PD1^hi^ CD200^low^ signature had a certain immuno‐effecting function similar to that previously reported,^42^ whereas the CD4^+^ Tex2 cluster, marked by high expression of PD1 and CD200, was in a complete exhaustion state.

### 3.2 The PD1^hi^ CD200^hi^ CD4^+^ exhausted T cells correlate with response to ICI

**FIGURE 2:**
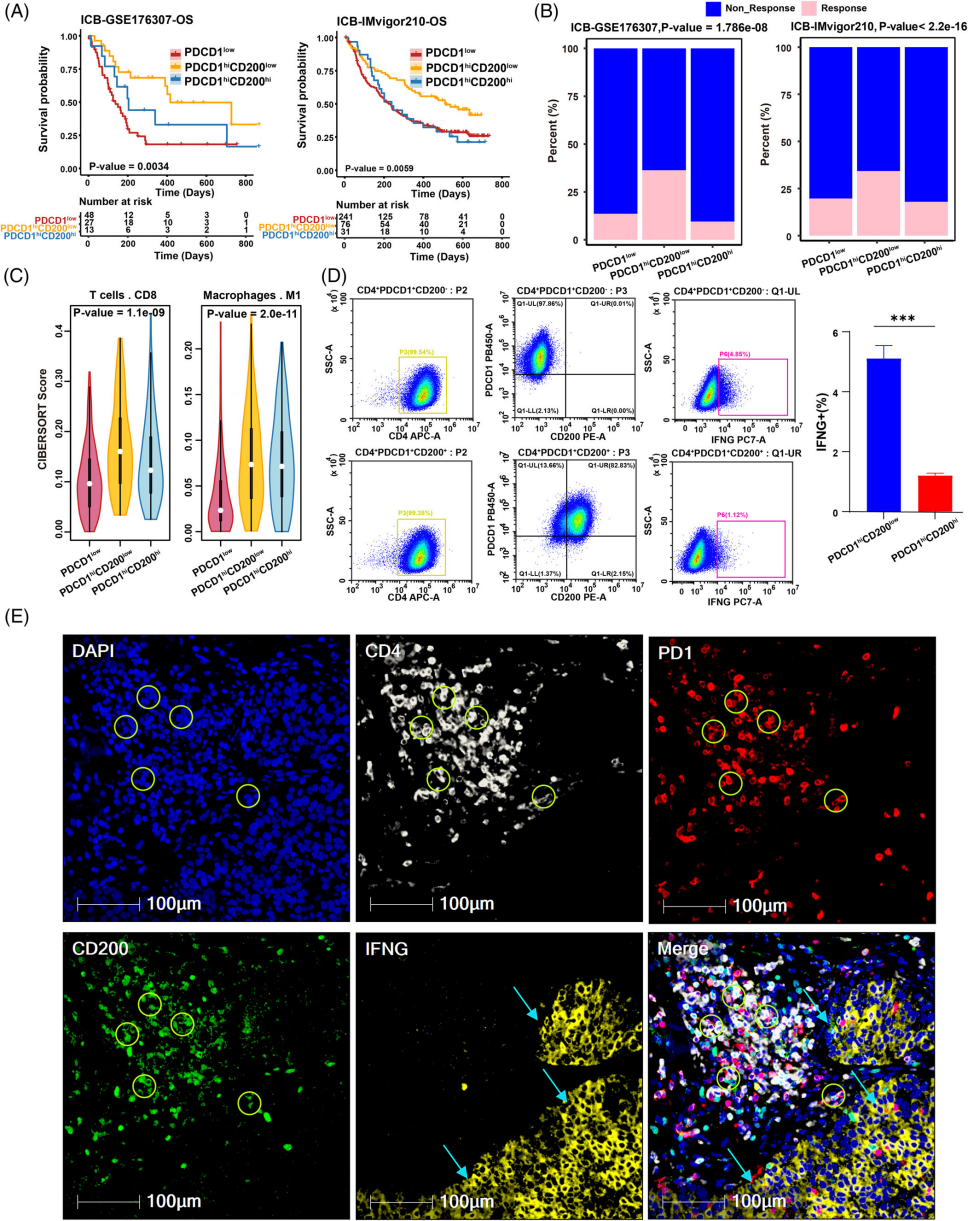
The PD‐1^hi^ CD200^hi^ CD4^+^ exhausted T cells predict a poor response to immunotherapy: (A) overall survival of patients with immunotherapy in PDCD1^low^, PDCD1^hi^ CD200^low^ and PDCD1^hi^ CD200^hi^ groups in the GSE176307 and IMvigor210 cohorts; (B) barplots showing the proportion of responders among PDCD1^low^, PDCD1^hi^ CD200^low^ and PDCD1^hi^ CD200^hi^ groups in the GSE176307 and IMvigor210 cohorts; (C) violin plots showing the estimation of the abundance of CD8 T cells and M1 macrophages in the PDCD1^low^, PDCD1^hi^ CD200^low^ and PDCD1^hi^ CD200^hi^ groups using CIBERSORT algorithm; (D) the expression of IFN‐γ between the PD‐1^hi^ CD200^low^ CD4^−^ T cells and PD‐1^hi^ CD200^hi^ CD4^−^ T cells was measured by intracellular flow cytometry. Summary data of IFN‐γ staining (right) are presented as mean ± SD (*n* = 3), **p < 0.01, ***p <0.001; (E) multiplex immunofluorescence staining was performed for DAPI (blue), CD4 (white), PD1 (red), CD200 (green) and IFNG (yellow). The circles represent the PD1^hi^ CD200^hi^ CD4^+^ exhausted T cells. The arrows represent the IFNG.

It is well known that rejuvenating exhausted T cells is an important research direction of cancer immunotherapy.^43^ In light of this, we investigated whether the PDCD1^hi^ CD200^hi^ group is related to the immunotherapeutic response in bladder cancer. Compared with the PDCD1^hi^ CD200^hi^ and PDCD1^low^ groups, the PDCD1^hi^ CD200^low^ group had the best survival rate and was most sensitive to immunotherapy based on the two immunotherapy cohorts GSE176307 and IMVigor210 (Figure 2A,B; Table S5; Figure S2A,C). Similar results were also obtained in the extended dataset of NSCLC (GSE135222), gastric cancer (PRJEB25780) and melanoma (GSE91062, phs000452) cohorts (Figure S2D–H). There was a strong indication from the above results that patients in the PDCD1^hi^ CD200^low^ group could benefit from immunotherapy, whereas those in the PDCD1^hi^ CD200^hi^ or PDCD1^low^ group appeared to be resistant to immunotherapy.

In terms of immunocyte infiltration, more CD8^+^ T cell infiltration was found in the PDCD1^hi^ CD200^low^ group than in the PDCD1^hi^ CD200^hi^ and PDCD1^low^ groups by employing the CIBERSORT algorithms. Moreover, more infiltration of M1 macrophage cells (Figure 2C, Table S6) and cytotoxic lymphocyte infiltration (Figure S3A) were found in the PDCD1^hi^ CD200^hi^ and PDCD1^hi^ CD200^low^ groups compared with those in the PDCD1^low^ group. In addition, a striking finding in the single‐cell RNA sequencing data was that the effector T‐cell molecules IFNG, GZMA, and GZMB were substantially expressed in the PDCD1^hi^ CD200^low^ group (Figure S3B–D). To further confirm these findings, flow cytometry was used to estimate IFNG expression in three bladder cancer samples, and the results revealed that PD1^hi^ CD200^low^ CD4^+^ exhausted T cells showed significantly higher expression of IFNG (Figure 2D). Immunohistochemistry staining in bladder cancer tissues further validated that strong staining of IFNG was observed near the tumour borders rather than around PD1^hi^ CD200^hi^ CD4^+^ exhausted T cells (Figure 2E; Figure S3E). Among the CD4^+^ T‐cell clusters, Th1 cells are characterized by the secretion of IFNG, which activates macrophages and CD8^+^ T cells.^44^, ^45^ Overall, our findings suggest that compared with PD1^hi^ CD200^low^ CD4 exhausted T cells, PD1^hi^ CD200^hi^ CD4 exhausted T cells may contribute to immune tolerance, and patients with high proportions of this subcluster often fail to respond to immunotherapy.

### 3.3 PD1^hi^ CD200^hi^ CD4^+^ exhausted T cells promote angiogenesis and recruit tip cells through the uridine triphosphate (UTP)/P2RY6 axis

**FIGURE 3:**
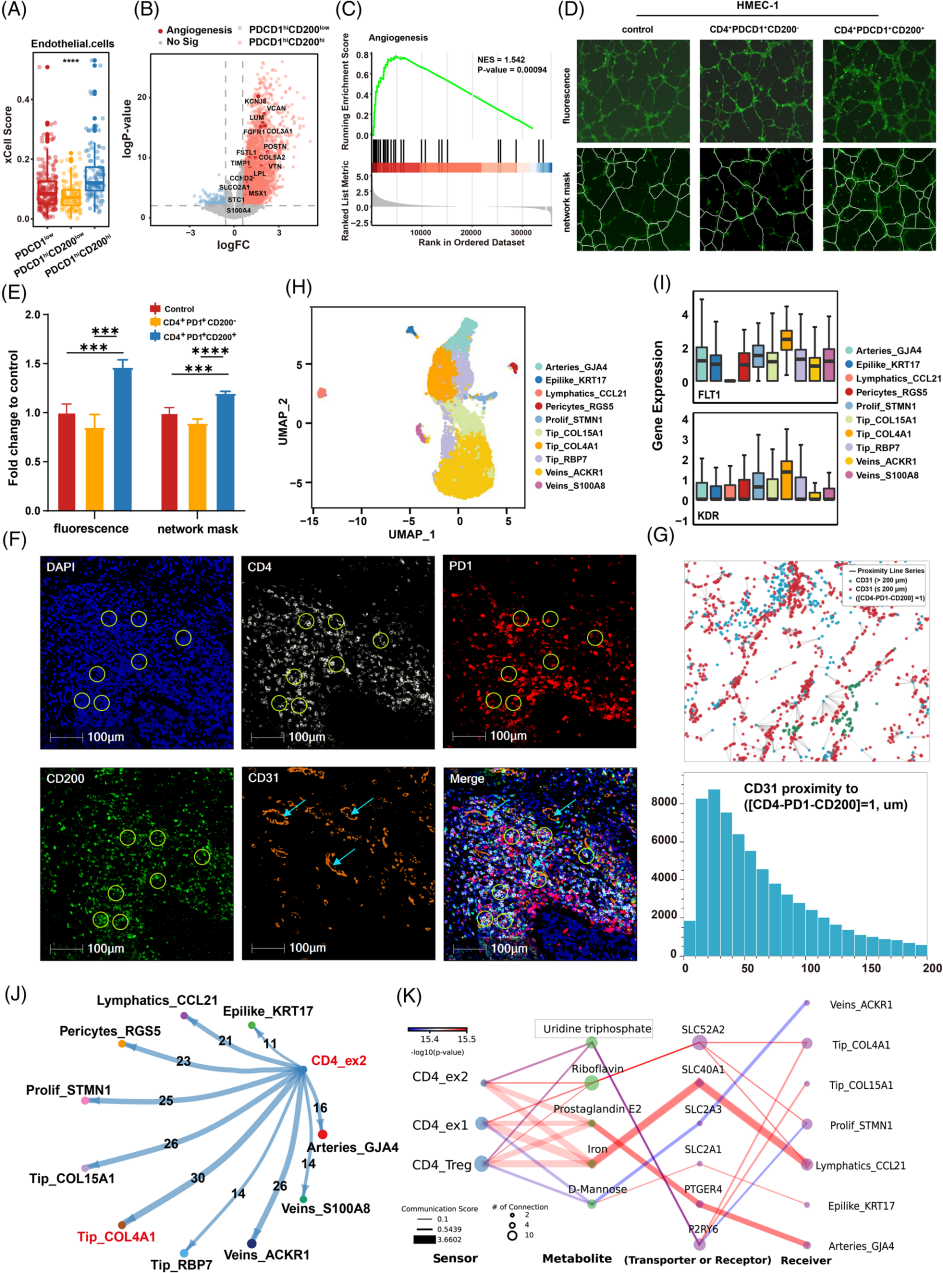
The PD‐1^hi^ CD200^hi^ CD4 exhausted T cells recruit tip cells to promote angiogenesis: (A) boxplot showing that estimation of the abundance of endothelial cells in the PDCD1^low^, PDCD1^hi^ CD200^low^ and PDCD1^hi^ CD200^hi^ groups using xCell_counter algorithm; (B) volcano map showing differentially expressed genes between PDCD1^hi^ CD200^hi^ and PDCD1^hi^ CD200^low^ groups (PDCD1^hi^ CD200^hi^: logFC >0.3 and log *p*‐value >2; PDCD1^hi^ CD200^low^: logFC < −0.3 and log *p*‐value >2). Angiogenesis pathway‐related genes were highlighted; (C) gene set enrichment analysis (GSEA) showing that the angiogenesis pathway was overrepresented in PDCD1hi CD200hi groups; (D and E) fluorescence micrograph of tube formation by HMEC1 (top) and network mask (bottom). Branch points and capillary length were analysed to evaluate angiogenic activity. The bars represent mean ± SD (*n* = 3), **p <0.01, ***p < 0.001; (F) multiplex immunofluorescence staining was performed for DAPI (blue), CD4 (white), PD1 (red), CD200 (green) and CD31 (orange). The circles represent the PD1^hi^ CD200^hi^ CD4^+^ exhausted T cells. The arrows represent the CD31; (G) spatial distribution of CD31^+^ cells around PD‐1^hi^ CD200^hi^ CD4^−^ T cells. The histogram showing the distribution of distances from each the PD‐1^hi^ CD200^hi^ CD4^−^ T cells the nearest CD31^+^ cell; (H) the UMAP plot showing the clusters of endothelial cells. Each dot indicates a single cell; (I) the boxplot showing the angiogenesis‐related genes expression in subclusters of endothelial cells; (J) the aggregated cell–cell communication analysis of CD4_ex2 and endothelial cells in BLCA by CellChat; (K) a flow diagram showing the information flow of metabolite–sensor communications from CD4^+^ T cells to endothelial cells through metabolites and sensors. The size of dots represents the number of connections. The lines connect the sender, metabolite, sensor and receiver. The colour of the line indicates the −log10(*p*‐value), and the width of lines represents the communication score.

To explore the mechanism by which the PDCD1^hi^ CD200^hi^ Group T cells reduce immunotherapy efficacy and patient survival, xCell algorithm analysis was performed using the TCGA dataset of 406 BLCA patients. The results revealed a significant increase in the number of endothelial cells in the PDCD1^hi^ CD200^hi^ group, indicating its role in promoting angiogenic activity (Figure 3A; Table S7). As angiogenesis is one of the ways that tumours are able to evade detection by the immune system,^46^ we hypothesized that PD1^hi^ CD200^hi^ CD4^+^ exhausted T cells might contribute to immunotherapy resistance by promoting angiogenesis. In line with expectations, differential gene expression profiles across the PDCD1^hi^ CD200^low^, PDCD1^hi^ CD200^hi^ and PDCD1^low^ groups indicated that the PDCD1^hi^ CD200^hi^ group exhibited the highest levels of angiogenesis‐related genes (Figure 3B; Figure S4A; Table S8). GSEA revealed that PDCD1^hi^ CD200^hi^ group was enriched in the angiogenesis pathway (Figure 3C; Table S9). GO biological pathway enrichment analysis also indicated that the PDCD1^hi^ CD200^hi^ group was predominantly related to the regulation of angiogenesis (Figure S4B). Next, we evaluated the proangiogenic potency of the PDCD1^hi^ CD200^hi^ group in vitro. HMEC‐1 cells (human microvascular endothelial cells) were cocultured with PD1^hi^ CD200^low^ T cells or PD1^hi^ CD200^hi^ T cells for 48 h and then used to assess tube formation in vitro. As revealed by fluorescence microscopy images of the tube formation assays, cocultivation with the PD1^hi^ CD200^hi^ group increased the capillary length and number of branch points of HMEC1 cells (Figure 3D,E). Next, we used multiplex immunofluorescence (CD4, PD1, CD200, CD31 and DAPI) to explore the spatial distance between the PD1^hi^ CD200^hi^ CD4 exhausted T cells and CD31, a marker for specific vascular endothelial cells (Figure 3F). Proximity analysis revealed that CD31^+^ endothelial cells were more abundant around PD1^hi^ CD200^hi^ CD4^+^ exhausted T cells than around PD1^hi^ CD200^low^ CD4^+^ exhausted T cells, indicating their proangiogenic potential (Figure 3G; Figure S4C). Given the above results, we speculated that PD1^hi^ CD200^hi^ CD4^+^ exhausted T cells can induce angiogenesis.

To gain insight into the underlying molecular mechanisms of the proangiogenic effect of PD1^hi^ CD200^hi^ CD4^+^ exhausted T cells, we created an endothelial cell atlas with single‐cell RNA‐Seq data on 9801 endothelial cells derived from tumour tissue and adjacent normal tissues of nine bladder cancer patients (Table S10). Endothelial cells were mainly found in tumour tissues, showing little heterogeneity between patients (Figure S4D,E). Ten endothelial cell clusters with distinct gene expression patterns were identified in the dataset (Figure 3H; Figure S4F; Table S11). Among the clusters, Tip_COL4A1 showed the highest expression levels of the angiogenesis‐promoting genes FLT1, KDR, CD93 and NRP1 (Figure 3I; Figure S4G). FLT1 and KDR are receptors of VEGF, which is the most important factor in promoting angiogenesis in tumours.^47^, ^48^ In addition, COL4A1 was also shown to play a major role in promoting neovascular sprouting.^49^ As shown above, Tip_COL4A1 seemed to have potent angiogenic activity. Notably, CellChat analysis revealed that the Tip_COL4A1 cluster communicates most frequently with PD1^hi^ CD200^hi^ CD4 exhausted T cells among the 10 endothelial cell clusters (Figure 3J). When applying the MEBOCOST algorithm on the CD4 Tex2 cluster as well as endothelial cells, we found that the Tip_COL4A1 cluster had the largest number of communications (133 events) compared to that of other endothelial cells as receiver cells (Figure S4H). In addition, among all metabolite‐sensor partners, uridine triphosphate (UTP) and its transporter P2RY6 were found to mediate communication between CD4 Tex2 cells and Tip_COL4A1 endothelial cells (Figure 3K). Furthermore, a higher expression of P2RY6 predicted worse survival in patients with bladder cancer in the TCGA database (Figure S4I). Thus, we suggest that PD1^hi^ CD200^hi^ CD4 exhausted T cells might induce angiogenesis and recruit Tip_COL4A1 endothelial cells through the UTP/P2RY6 axis.

### 3.4 Crosstalk between PD1^hi^ CD200^hi^ CD4^+^ exhausted T cells and tumour cells through AXL–GAS6 promotes EMT in bladder cancer

**FIGURE 4:**
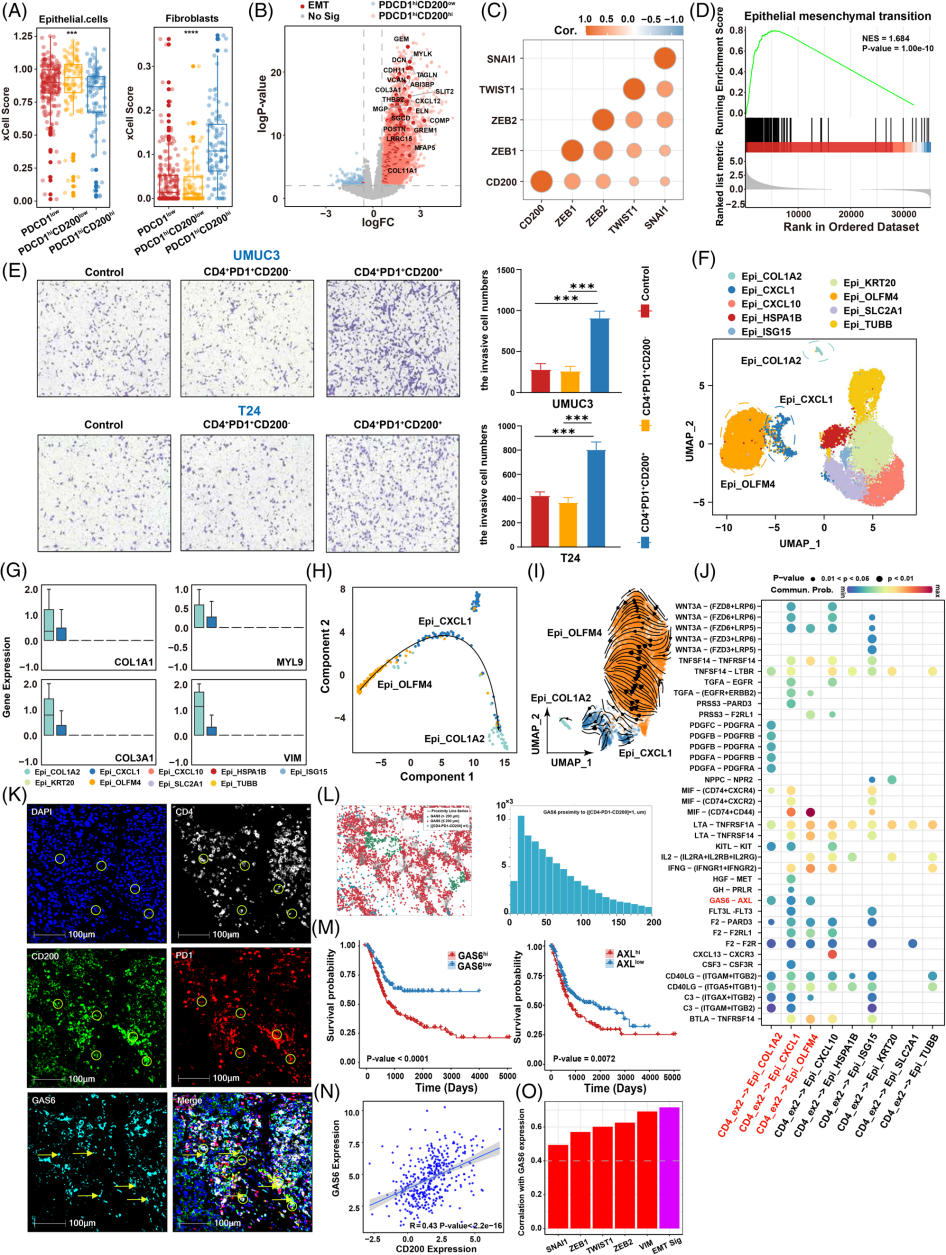
The PD1^hi^ CD200^hi^ CD4 exhausted T cells promote epithelial–mesenchymal transition (EMT): (A) boxplot showing that estimation of the abundance of epithelial and fibroblast cells in the PDCD1^low^, PDCD1^hi^ CD200^low^ and PDCD1^hi^ CD200^hi^ groups using xCel algorithm; (B) volcano map showing differentially expressed genes between PDCD1^hi^ CD200^hi^ and PDCD1^hi^ CD200^low^ groups (PDCD1^hi^ CD200^hi^: logFC > 0.3 and log *p*‐value >2; PDCD1^hi^ CD200^low^: logFC < −0.3 and log *p*‐value >2). The EMT pathway‐related genes were highlighted; (C) correlation of CD200 expression with the expression of transcription factor of EMT. The colour indicates the Spearman correlation coefficient; (D) gene set enrichment analysis (GSEA) showing that the EMT pathway was overrepresented in PDCD1^hi^ CD200^hi^ groups; (E) the invasion of T24 and UMUC3 cells was detected by transwell assay after co‐cultured with the PD1^hi^ CD200^hi^ CD4^+^ or PD1^hi^ CD200^low^ CD4^+^ exhausted T cells. Results are presented as mean ± SD (*n* = 3), **p < 0.01, ***p < 0.001; (F) the UMAP plot showing the clusters of epithelial cells. Each dot indicated a single cell. Colour‐coded for the cell type; (G) the boxplot showing the EMT‐related genes expression in clusters of epithelial cells; (H) analysis of simulated differentiation trajectories of three epithelial cell subclusters (Epi_CXCL1, Epi_OLFM4, and Epi_COL1A2) in BLCA. Each dot corresponds to a cell, and each colour represents an epithelial cell subcluster; (I) the RNA velocity analysis graph reflected the evolutionary relationship among the three epithelial cell subclusters; (J) the significantly enriched ligand–receptor pairs between epithelial cells and CD4_ex2 subcluster T cells in primary tumour; (K) multiplex immunofluorescence staining was performed for DAPI (blue), CD4 (white), PD1 (green), CD200 (red) and GAS6 (lawngreen). The circles represent the PD1^hi^ CD200^hi^ CD4^+^ exhausted T cells. The arrows represent the GAS6; (L) spatial distribution of GAS6^+^ cells around PD‐1^hi^ CD200^hi^ CD4^+^ T cells, DAPI (blue), CD4 (white), PD1 (red), CD200 (green) and GAS6 (lawngreen); (M) overall survival between the high and low expressions of GAS6 or AXL groups in The Cancer Genome Atlas (TCGA)‐BLCA; (N) scatter plot showing the correlation between the expression of CD200 and GAS6 in TCGA‐BLCA; (O) barplot showing the correlation between the expression of EMT transcription factor gene, EMT signature and GAS6 in TCGA‐BLCA.

The xCell algorithms on BLCA data in the TCGA database demonstrated not only a decrease in epithelial cells but also an increase in fibroblasts in the PDCD1^hi^ CD200^hi^ group, indicating a possible EMT phenotype (Figure 4A). EMT has been associated with cancer invasion and immune resistance.^50^ Consequently, we hypothesized that PD1^hi^ CD200^hi^ CD4^+^ exhausted T cells may promote EMT in bladder cancer cells, thus contributing to immunotherapy resistance. Consistent with our hypothesis, a higher expression of the EMT‐related genes CDH11 and DCN was found in the PDCD1^hi^ CD200^hi^ group than in the PDCD1^hi^ CD200^low^ group and other CD4^+^ T‐cell subclusters (Figure 4B; Figure S5A). We also found that CD200 was positively correlated with the EMT‐related transcription factors ZEB1, ZEB2, TWIST1 and SNAI1 (Figure 4C). GSEA showed that the EMT and myogenic pathways were enriched in the PDCD1^hi^ CD200^hi^ group (Figure 4D; Figure S5B). Furthermore, an invasion experiment was performed using the bladder cancer cell Lines T24 and UMUC3 cells cocultured with exhausted T cells. The results showed that the invasive capacity of T24 and UMUC3 cells significantly increased after coincubation with PD1^hi^ CD200^hi^ CD4^+^ exhausted T cells (Figure 4E). The above results indicated that PD1^hi^ CD200^hi^ CD4^+^ exhausted T cells induce EMT and enhance the invasion of bladder cancer cells. Next, we created an epithelial cell atlas using 24 172 epithelial cells derived from tumour tissue and adjacent normal tissues from 8 BLCA patients (Table S12). The identified epithelial cells were mainly derived from tumour tissues (Figure S5C). Nine different epithelial cell clusters with unique gene expression patterns were identified using unsupervised clustering (Figure 4F). High expression of epithelial‐related genes, such as KRT8, KRT18 and EPCAM, was found in all clusters (Figure S5D; Table S13), proving that all cells can be identified as epithelial cells. Surprisingly, high levels of COL1A1, MYL9, COL3A1 and vimentin were found in the clusters Epi_OLFM4 and Epi_CXCL1, indicating that the cells might be fibroblast‐like epithelial cells (Figure 4G). Additionally, the clusters Epi_OLFM4 and Epi_CXCL1 expressed high levels of CD44, CD55, KLF4 and ALDH1A1, suggesting that they are stem‐like cells (Figure S5E). The above results revealed that the epithelial cell clusters Epi_OLFM4 and Epi_CXCL1 may have undergone EMT and transitioned into invasive mesenchymal cells. The results from trajectory analysis, RNA velocity analysis and PAGA analysis showed that Epi_COL1A2 developed from Epi_OLFM4 and Epi_CXCL1 was the intermediate transition stage (Figure 4H,I; Figure S5F). Next, CellChat analysis revealed that the receptor‒ligand pair GAS6–AXL was significantly enriched in CD4 ex2‐Epi_OLFM4, CD4 ex2‐Epi_CXCL1 and CD4 ex2‐Epi_COL1A2 (Figure 4J). Moreover, multiplex immunofluorescence (CD4, PD1, CD200, GAS6 and DAPI) was performed to investigate the spatial distance between the PD1^hi^ CD200^hi^ CD4 exhausted T cells and GAS6. The proximity analysis showed plentiful GAS6 in the vicinity of the PD1^hi^ CD200^hi^ CD4^+^ exhausted T cells compared to the PD1^hi^ CD200^low^ CD4^+^ exhausted T cells (Figure 4K,L; Figure S6A). Thus, we proposed that PD1^hi^ CD200^hi^ CD4 exhausted T cells and malignant cells may communicate via AXL–GAS6 signalling in bladder cancer. The Kaplan‒Meier survival analysis showed that the higher expression of GAS6 was associated with worse OS, progression‐free survival and platinum‐free interval in 406 BLCA patients in the TCGA dataset (Figure 4M; Figure S7A). A similar trend was found in the Kaplan‒Meier survival analysis for BLCA patients in the TCGA dataset with high levels of AXL (Figure 4M; Figure S7B). High GAS6 expression was also associated with more advanced N stage (Figure S7C). In addition, the correlation analysis showed a significant and positive correlation between CD200 and GAS6 (*R* = 0.43, Figure 4N). The PDCD1^hi^ CD200^hi^ group exhibited a higher level of GAS6 expression than the PDCD1^low^ and PDCD1^hi^ CD200^low^ groups (Figure S7D). Furthermore, we also found that GAS6 was positively related to the EMT‐related transcription factors ZEB1, ZEB2, TWIST1, SNAI1 and vimentin (Figure 4O). GSEA results also revealed that GAS6 expression significantly and positively correlated with the EMT signatures (Figure S7E,F). The results above indicated that the GAS6–AXL axis might interact with the PD1^hi^ CD200^hi^ CD4 exhausted T cells, trigger EMT and promote bladder cancer progression.

### 3.5 PD1^hi^ CD200^hi^ CD4^+^ exhausted T cells negatively correlate with the response to ICIs and positively recruit vascular cells in vivo

**FIGURE 5:**
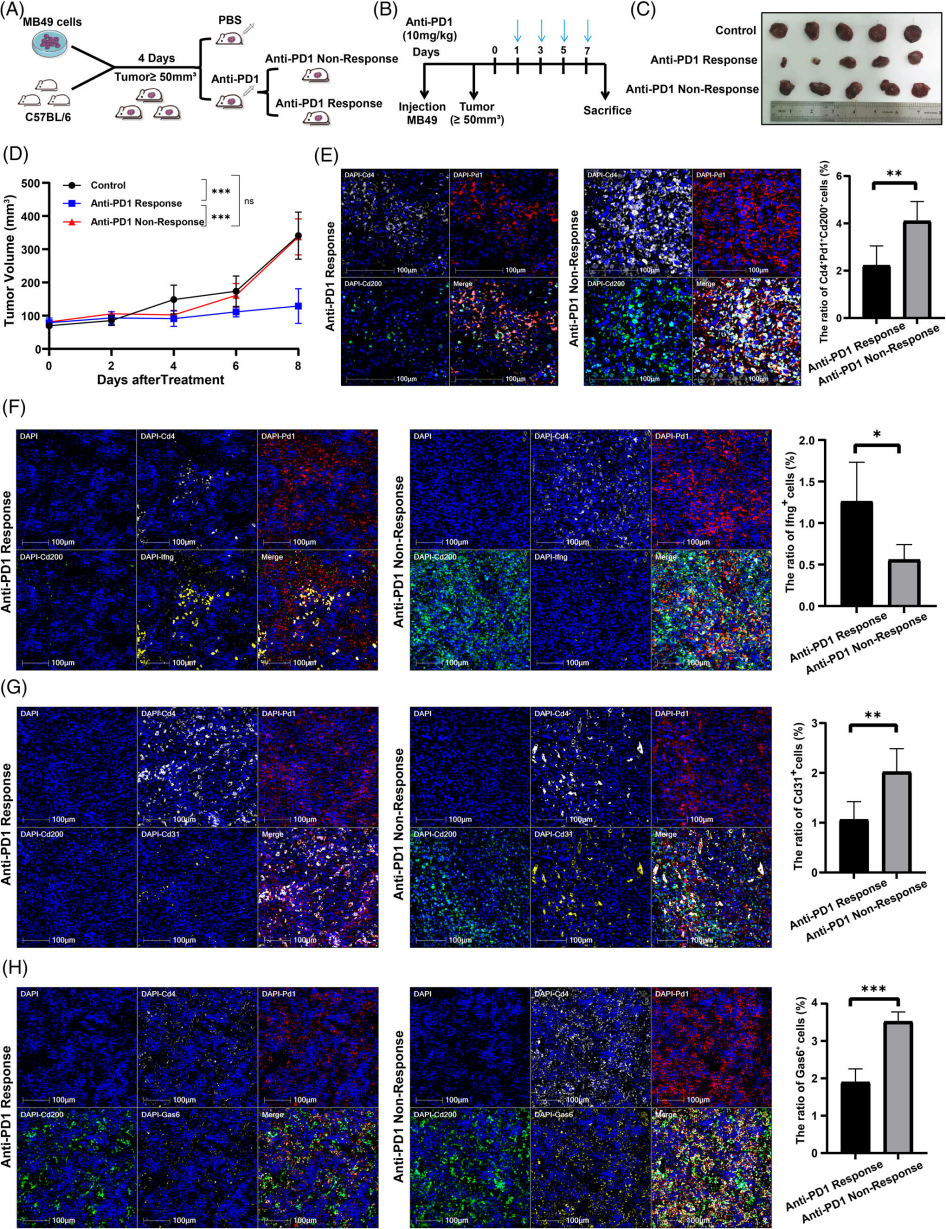
PD1^hi^ CD200^hi^ CD4^+^ exhausted T cells negatively correlate with the response to ICIs and positively recruit vascular cells in vivo: (A) schematic diagram of mice models; (B) schematic diagram of administration cycles in mice; (C) photographs of tumours dissected out from the three mice groups; (D) tumour volumes were measured once every days, and growth curves of tumours were shown; (E) multiplex immunofluorescence staining was performed for DAPI (blue), Cd4 (white), Pd1 (red) and Cd200 (green); each stain was shown separately and merged. Histograms show the percentages of Pd1hi Cd200hi Cd4^+^ exhausted T cells in anti‐PD1‐responsive and non‐responsive groups; (F) multiplex immunofluorescence staining was performed for DAPI (blue), Cd4 (white), Pd1 (red) and Cd200 (green); each stain was shown separately and merged. Histograms show the percentages of Pd1hi Cd200hi Cd4^+^ exhausted T cells in anti‐PD1‐responsive and non‐responsive groups; (G) multiplex immunofluorescence staining was performed for DAPI (blue), Cd4 (white), Pd1 (red), Cd200 (green) and Ifng (yellow); each stain was shown separately and merged. Histograms show the percentages of Ifng in anti‐PD1‐responsive and non‐responsive groups; (G) multiplex immunofluorescence staining was performed for DAPI (blue), Cd4 (white), Pd1 (red), Cd200 (green) and Cd31 (yellow); each stain was shown separately and merged. Histograms show the percentages of Cd31 in anti‐PD1‐responsive and non‐responsive groups; (H) multiplex immunofluorescence staining was performed for DAPI (blue), Cd4 (white), Pd1 (red), Cd200 (green) and Gas6 (yellow); each stain was shown separately and merged. Histograms show the percentages of Gas6 in anti‐PD1‐responsive and non‐responsive groups.

To verify the function of PD1^hi^ CD200^hi^ CD4^+^ exhausted T cells in vivo, we subcutaneously implanted MB49 bladder cancer cells into immunocompetent mice and treated mice every 2 days with anti‐PD1 antibody when tumours were 50 mm^3^ in size (Figure 5A,B). According to the tumour growth curve, mice were divided into anti‐PD1‐responsive and nonresponsive groups (Figure 5C,D). Multiplex immunofluorescence was performed to determine the infiltration of PD1^hi^ CD200^hi^ CD4^+^ exhausted T cells in mice divided into anti‐PD1‐responsive and nonresponsive groups, which showed that the nonresponsive groups exhibited higher levels of Pd1^hi^ Cd200^hi^ Cd4^+^ exhausted T cells than the anti‐PD1‐responsive groups (Figure 5E). Next, multiplex immunofluorescence revealed that Pd1^hi^ Cd200^hi^ Cd4^+^ exhausted T cells secreted less Ifng than Pd1^hi^ Cd200^low^ Cd4^+^ exhausted T cells, and the nonresponsive groups had higher levels of Ifng (Figure 5F). Next, as a result of multiplex immunofluorescence, Pd1^hi^ Cd200^hi^ Cd4^+^ exhausted T cells were found to produce more Cd31 than Pd1^hi^ Cd200^low^ Cd4^+^ exhausted T cells, and Cd31 levels were higher in the nonresponsive group than in the anti‐PD1 group (Figure 5G). In addition, multiplex immunofluorescence revealed that Pd1^hi^ Cd200^hi^ Cd4^+^ exhausted T cells produced more Gas6, and Gas6 levels were higher in the nonresponsive group than in the anti‐PD1 group (Figure 5H).

### 3.6 METTL3‐mediated m6A modification enhances GAS6 expression in bladder cancer cells

**FIGURE 6:**
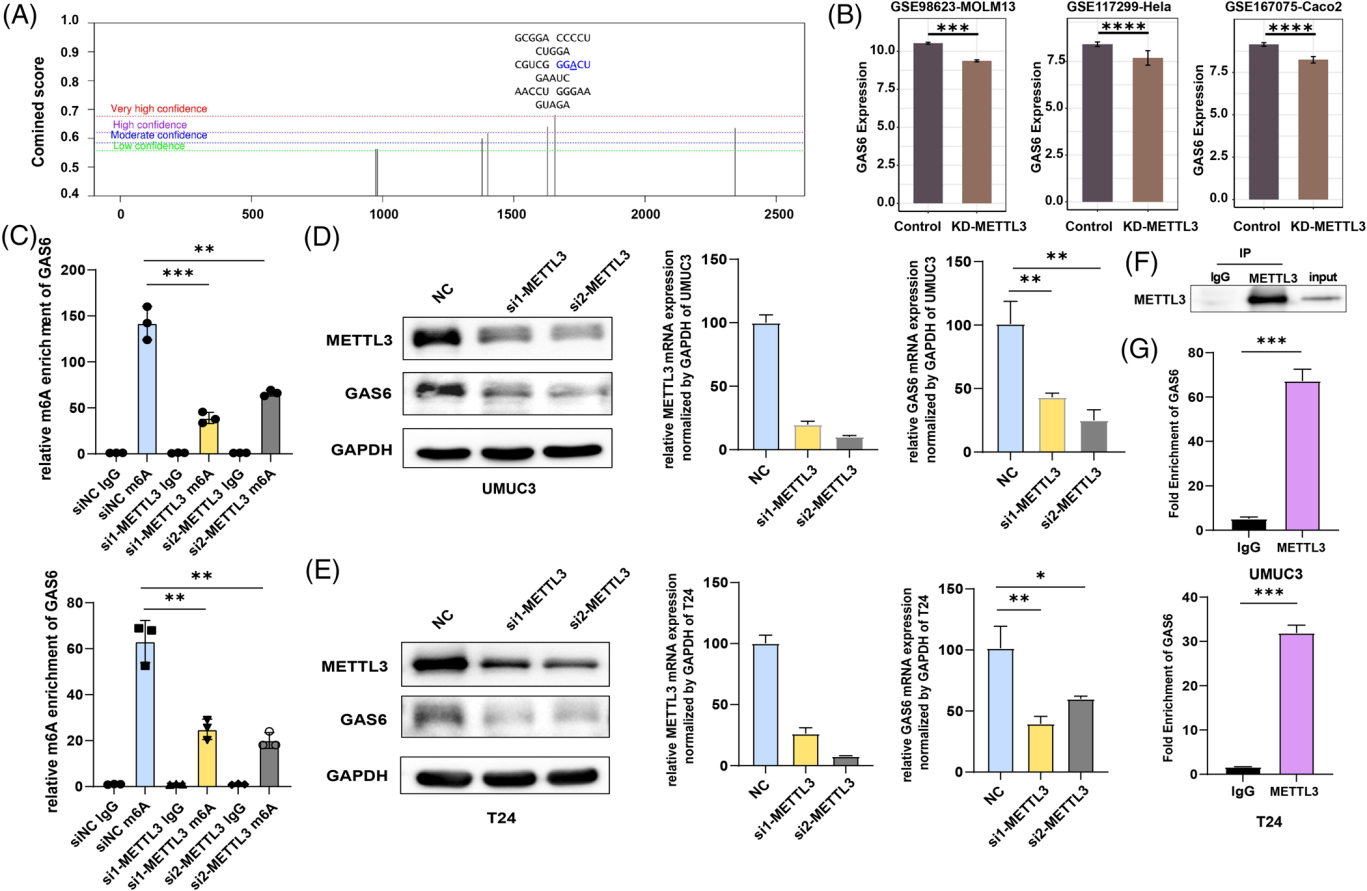
METTL3‐mediated m6A modification enhances GAS6 expression in BLCA cells: (A) the prediction of m6A RNA modification sites in GAS6; (B) GAS6 gene expression between the control and knockout‐METTL3 groups in three cohorts of knockout‐METTL3; (C) methylated RNA immunoprecipitation (RIP) and real‐time polymerase chain reaction analysis of the m6A levels of GAS6; (D and E) the protein levels and the relative mRNA levels of GAS6 after co‐transfection with METTL3 Knockdown (si‐METTL3) into UMUC3 (D) and T24 cells (E); (F) western blotting was performed with METTL3 antibodies to show immunoprecipitation efficiency; (G) RIP assay for the enrichment of GAS6 with METTL3.

The most abundant base modification in eukaryotic mRNA, N6‐methyladenosine (m6A), is known to modulate gene expression in various cancer types.^51^, ^52^ Based on the SRAMP (sequence‐based m6A modification site predictor) algorithm,^53^ we identified six m6A sites with high scores in the 3′UTR of GAS6 mRNA (Figure 6A). We also discovered that the knock‐down of METTL3 in MOLM13, HeLa and Caco2 cell lines can downregulate the mRNA expression of GAS6 using three GSE datasets (Figure 6B). Based on the above results, we proposed that METTL3 may affect the expression of GAS6 by regulating the level of m6A modification. To validate this hypothesis, we first performed gene‐specific m6A methylated RIP qRT‐PCR analysis in bladder cancer cells transfected with or without METTL3 siRNA. As expected, METTL3 knock‐down significantly reduced the m6A enrichment of GAS6 (Figure 6C). The findings suggested that METTL3 could directly regulate the m6A methylation of GAS6 mRNA. Next, GAS6 expression at both RNA and protein levels was reduced after METTL3 knock‐down in the bladder cancer cell Lines T24 and UMUC3 (Figure 6D,E). In addition, RIP assays demonstrated direct interactions between GAS6 mRNA and METTL3 (Figure 6F,G). In conclusion, these findings suggested that METTL3 could regulate GAS6 expression in an m6A‐dependent manner in bladder cancer cells.

## 4 DISCUSSION

CD4^+^ T‐cell subsets with different functions are found in a wide range of cancers.^54^, ^55^, ^56^ To date, a pancancer T‐cell atlas has been established, and two types of CD8^+^ exhausted T cells were identified.^57^ However, knowledge on CD4^+^ exhausted T cells is still scarce. In the pancancer CD4^+^ T‐cell atlas, we found two different clusters of CD4^+^ exhausted T cells, CD4_Tex1 and CD4_Tex2, defined according to PD‐1 and CD200 expressions. In addition, our findings revealed that CD4_Tex1 cells have Th17‐like gene expression, whereas CD4_Tex2 cells have Tfh‐like gene expression. CD200 (also known as OX2), a transmembrane glycoprotein of the immunoglobulin supergene family, is mainly expressed in several types of cancer cells, endothelial cells and activated immune cells, whereas its receptor (CD200R) is mostly expressed in monocytes/myeloid cells and T lymphocytes.^58^, ^59^, ^60^ As a newly emerging immune checkpoint molecule, CD200 mediates immunoregulatory signals by binding to CD200Rs to suppress the antitumour immune response and regulate immune tolerance, adhesion, differentiation and chemotaxis.^61^, ^62^ In CD4^+^ T cells, the CD200–CD200R pathway is important for regulating their differentiation. The imbalance of Tregs/Th17 cells can be caused by the CD200–CD200R signalling pathway, which induces Treg generation and prevents Th17 differentiation, thus resulting in immune tolerance.^63^, ^64^ Moreover, the activation of the CD200/CD200R signalling pathway is also a key contributor to the Treg phenotype.^65^, ^66^ In light of the above studies, we hypothesized that PD1^hi^ CD200^hi^ CD4^+^ exhausted T cells may have the traits of Tregs. Next, our results indicated that patients with high proportions of PD1^hi^ CD200^low^ CD4^+^ exhausted T cells had an excellent prognosis following immunotherapy, whereas those with high proportions of PD1^hi^ CD200^hi^ CD4^+^ exhausted T cells demonstrated immune tolerance. Furthermore, in addition to the high infiltration of CD8 T cells and cytotoxic lymphocytes, PD1^hi^ CD200^low^ CD4^+^ exhausted T cells are also shown to consistently produce IFNG, GZMA and GZMB despite being exhausted.^67^ Therefore, we proposed that PD1^hi^ CD200^low^ CD4^+^ exhausted T cells in the TME might induce the expansion and activation of intratumoural CD8^+^ T cells, thus leading to better immunological responses in bladder cancers patients. In contrast, Tregs are known to represent the majority of CD4^+^ T cells that inhibit immunity.^68^, ^69^ As the CD200–CD200R signalling pathway can contribute to the generation of Tregs, we speculate that the characteristics of PD1^hi^ CD200^hi^ CD4^+^ T cells may be similar to those of Tregs. Further exploration is necessary on this point.

As determined by xCell algorithms, the PDCD1^hi^ CD200^hi^ group exhibited an increased number of endothelial cells, which is associated with angiogenesis. The expression of the angiogenesis‐related genes KCNJ8 and VCAN was also higher in the PDCD1^hi^ CD200^hi^ group than in the other CD4^+^ T‐cell subclusters. Using in vitro experiments, we further proved that PD1^hi^ CD200^hi^ CD4^+^ exhausted T cells contribute to stimulating angiogenesis in bladder cancer. In the TME, angiogenesis plays a key role in facilitating immune tolerance by blocking cytotoxic T lymphocytes from entering.^70^ Thus, one of the reasons why PD1^hi^ CD200^hi^ CD4^+^ exhausted T cells are resistant to immunotherapy may lie in their ability to induce angiogenesis. Numerous studies have shown that Tregs promote angiogenesis.^71^, ^72^, ^73^, ^74^, ^75^, ^76^, ^77^ In detail, Tregs have been shown to increase VEGFA levels directly, thereby stimulating angiogenesis.^78^ Through their release of paracrine factors (e.g. IL‐10 and amphiregulin) and modulation of macrophage function, Tregs can indirectly influence angiogenesis beyond their VEGF‐mediated effects.^79^, ^80^ The results of the above research confirmed our conjecture that PD1^hi^ CD200^hi^ CD4^+^ T cells and Tregs may form a transformation relationship in some contexts. In the next step, we employed scRNA‐seq to comprehensively reveal novel cellular interactions between CD4^+^ exhausted T cells and endothelial cells at single‐cell resolution. Of all endothelial cell clusters, Tip_COL4A1 most frequently communicated with PD1^hi^ CD200^hi^ CD4^+^ exhausted T cells. Known as sprouting endothelial cells, Tip cells play a significant role in neovascularization, which is strongly affected by the tumour metabolic microenvironment.^81^, ^82^, ^83^ In light of this, we discovered that the metabolite‐sensor partner UTP‐P2YR6 was activated specifically in the cell interaction between PD1^hi^ CD200^hi^ CD4^+^ exhausted T cells and the Tip_COL4A1 subcluster. When stressed or inflamed, cells will increase the exocytotic release of UTP and then bind with P2YR6 to activate a series of downstream pathways, including inducing chemotaxis and migration and regulating vascular remodelling.^84^, ^85^, ^86^, ^87^, ^88^ Overall, we conclude that paracrine secretion of UTP by PD1^hi^ CD200^hi^ CD4 exhausted T cells can recruit the sprouting endothelial cells Tip_COL4A1 to initiate angiogenesis.

There is widespread recognition that EMT plays a critical role in immune resistance.^89^ An EMT phenotype with decreased epithelial cells and increased fibroblasts was demonstrated in the PDCD1^hi^ CD200^hi^ group by using xCell algorithms in this study. Moreover, the expression of the EMT transcription factors CDH11 and DCN was higher in the PDCD1^hi^ CD200^hi^ group than in the other CD4^+^ T‐cell subclusters. A significant and positive correlation was found between CD200 and the EMT‐related transcription factors ZEB1, ZEB2, TWIST1 and SNAI1 in TCGA data. The EMT pathway was enriched in the PDCD1^hi^ CD200^hi^ group by GSEA. Therefore, driving EMT in bladder cancer might be another mechanism by which PD1^hi^ CD200^hi^ CD4^+^ exhausted T cells promote immune escape in bladder cancer, in addition to inducing angiogenesis. Recent studies have indicated that Tregs can facilitate EMT by promoting the expression of β‐Catenin and TGF‐β in epithelial cells.^90^, ^91^, ^92^ Nonetheless, studies providing evidence of how other CD4^+^ T‐cell clusters regulate EMT are scarce. To discover the underlying mechanism, an atlas of epithelial cells was constructed using bladder cancer scRNA‐seq, and nine distinct epithelial cell subclusters were identified. Among them, the Epi_OLFM4 and Epi_CXCL1 subclusters showed EMT transition with a high expression of mesenchymal biomarkers. Interestingly, the GAS6–AXL axis was specifically enriched in the cell interaction between PD1^hi^ CD200^hi^ CD4^+^ exhausted T cells and Epi_OLFM4, Epi_CXCL1 and Epi_COL1A2. The GAS6/AXL signalling pathway has been shown to promote tumour cell proliferation and survival as well as EMT and immune evasion.^93^ As reported, AXL has major roles in the migration of cells during development and regulates cell‒cell communication among cancer cells, endothelial cells and immune cells by binding to its ligand GAS6.^94^, ^95^ In addition, GAS6 has a direct positive effect on the functions of Tregs mainly through its interaction with the AXL receptor.^96^ Evidence exists that GAS6 can induce AXL‐mediated chemotaxis.^97^ As expected, using six‐colour mHIC, we validated the result that GAS6 is capable of inducing the chemotaxis of PD1^hi^ CD200^hi^ CD4^+^ exhausted T cells. Considering the above studies, we hypothesized that malignant cells in bladder cancer may recruit PD1^hi^ CD200^hi^ CD4+ exhausted T cells via the GAS6/AXL axis, and that PD1^hi^ CD200^hi^ CD4^+^ exhausted T cells may promote EMT in bladder cancer cells. The mechanisms by which bladder cancer cells upregulate GAS6 expression are also discussed. m6A represents the most prevalent epigenetic modification of RNAs.^98^ There is evidence pointing to a regulatory role for m6A modification in GAS6 expression.^99^, ^100^ Additionally, we verified that m6A can regulate GAS6 expression through METTL3 in bladder cancer cells using immunoprecipitation experiments and in vitro experiments. Overall, the PD1^hi^ CD200^hi^ CD4^+^ T cells might be recruited and contribute to the EMT of bladder cancer cells through m6A‐mediated GAS6.

## 5 CONCLUSION

In this study, we constructed a pancancer CD4^+^ T‐cell atlas based on single‐cell RNA‐Seq data from patients with multiple cancers types and identified two clusters of PD1^hi^ CD200^low^ and PD1^hi^ CD200^hi^ CD4^+^ exhausted T cells in bladder cancer patients. More importantly, bladder cancer patients with high proportions of PD1^hi^ CD200^hi^ CD4^+^ exhausted T cells showed a worse response to immunotherapy than those with PD1^hi^ CD200^low^ CD4^+^ exhausted T cells. We created a single‐cell atlas of endothelial cells and revealed that PD1^hi^ CD200^hi^ CD4^+^ exhausted T cells might recruit tip endothelial cells and initiate angiogenesis through UTP and its transporter P2RY6. In addition, we also found that bladder cancer cells can recruit PD1^hi^ CD200^hi^ CD4^+^ exhausted T cells to promote EMT by enhancing m6A‐mediated GAS6. Finally, our findings suggest that immunotherapy combined with anti‐CD200 drugs might be a promising therapeutic option for bladder cancer patients with high proportions of PD1^hi^ CD200^hi^ CD4^+^ exhausted T cells. Therefore, this research provides a valuable insight into CD4^+^ exhausted T cells and their influence on immunotherapy. However, there are several limitations to our study. Further validation will be conducted in a prospective and multicentre randomized trial in the future (Graphical Abstract).

## CONFLICT OF INTEREST STATEMENT

The authors declare that they have no conflict of interest.

## Supporting information


**Figure S1 The PD1^hi^ CD200^hi^ CD4^+^ exhausted T cells in the tumour microenvironment: (A)** the UMAP plot showing pan‐CD4^+^ T cells coloured by tissue types. Each dot indicates a single cell. Colour‐coded for the tissue type; (B) the UMAP plot showing the CD4^+^ T cells labelling cancer types; (C) heat map showing the expression of signature genes in CD4 subclusters; (D) barplot showing the percentage of cancer types in each cluster of pan‐CD4 cells; (E) barplot showing the percentage of each cluster of pan‐CD4 cells in each dataset; (F) the UMAP plot showing the exhausted‐related genes (PDCD1, CTLA4, LAG3 and TIGIT) expression in pan‐CD4^+^ T cells atlas; (G) the boxplot showing the expression of Th17‐related and Tfh‐related gene in CD4_Tex1 and CD4_Tex2 clusters; (H) correlation of the expression of CD200 and CD4^+^ T‐cell signature genes (CD4, CD3D, CD3E and CD3G) in TCGA‐BLCA; (I) validation of PD1^hi^ CD200^low^ CD4^+^ T cells and PD‐1^hi^ CD200^hi^ CD4^+^ T cells by flow cytometry. BCC; basal cell carcinoma; BTCC, bladder transitional cell carcinoma; CCRCC, clear cell renal carcinoma; CRC, colorectal cancer; ESCC, oesophageal squamous cell carcinoma; HNSC, head and neck squamous cell carcinoma; Lung, lung cancer, NPC: nasopharyngeal carcinoma.

Supporting Information

**Figure S2 The PD1^hi^ CD200^hi^ CD4^+^ exhausted T cells predicted the poor response to immunotherapy in pancancer: (A–C)** overall survival of patients with immunotherapy in PDCD1^low^, PDCD1^hi^ CD200^low^ and PDCD1^hi^ CD200^hi^ groups in the GSE91061 and phs00052 (melanoma), and GSE135222 (NSCLC) cohorts; (D and E) boxplots showing the proportion of responders among PDCD1^low^, PDCD1^hi^ CD200^low^ and PDCD1^hi^ CD200^hi^ groups in the PRJEB25780 (STAD) and Braun_2020 (RCC) cohorts; (F) overall survival of patients with immunotherapy in PDCD1^hi^ CD200^low^ and PDCD1^hi^ CD200^hi^ groups in the IMvigor210 cohorts; (G and H) boxplots showing the proportion of responders between PDCD1^hi^ CD200^low^ and PDCD1^hi^ CD200^hi^ groups in the IMvigor210 and GSE176307 cohorts.

Supporting Information

**Figure S3 The expression of cytotoxic factors in the PD1^hi^ CD200^hi^ CD4^+^ exhausted T cells: (A)** violin plot showing the estimation of the abundance of cytotoxic lymphocyte in the PDCD1^low^, PDCD1^hi^ CD200^low^ and PDCD1^hi^ CD200^hi^ groups using CIBERSORT_counter algorithm; (B–D) the UMAP plot showing the expression of genes associated with T‐effector (IFNG, GZMA and GZMB) in pan‐CD4^+^ T cells; (E) spatial distribution of IFNG^+^ cells around PD‐1^hi^ CD200^hi^ CD4^+^ exhausted T cells.

Supporting Information

**Figure S4 The PD1^hi^ CD200^hi^ CD4^+^ exhausted T cells induced the angiogenesis in bladder cancer: (A)** volcano plot showing differentially expressed genes between PDCD1^hi^ CD200^hi^ and other (PDCD1^low^ and PDCD1^hi^ CD200^low^) groups (PDCD1^hi^ CD200^hi^: logFC > 0.3 and log *p*‐value >2; other: logFC < −0.3 and log *p*‐value >2). The angiogenesis pathway–related genes were highlighted; (B) barplot showing the pathway enrichment in PDCD1^hi^ CD200^hi^ group in TCGA‐BLCA; (C) multiplex immunofluorescence staining was performed for DAPI (blue), CD4 (white), PD1 (red), CD200 (green) and CD31 (orange); (D) the UMAP plot showing endothelial cells coloured by different tissue; (E) the UMAP plot showing endothelial cells coloured by each patient; (F) the heat map shows the top eight particular genes for each subcluster of endothelial cells; (G) the boxplot showing the angiogenesis‐related genes expressed in subclusters of endothelial cells; (H) barplot showing the number of communications for senders and receivers. The *x* axis is the subclusters of endothelial cells. The *y* axis is the number of communications. The orange and purple bars are the number of communications for sender and receiver cells, respectively; (I) disease‐free survival and progression‐free interval between the high and low expressions of P2RY6 groups in TCGA‐BLCA.

Supporting Information

**Figure S5 The PD1^hi^ CD200^hi^ CD4^+^ exhausted T cells promoted the epithelial–mesenchymal transition (EMT) in bladder cancer: (A)** volcano map showing differentially expressed genes between PDCD1^hi^ CD200^hi^ and other (PDCD1^low^ and PDCD1^hi^ CD200^low^) groups (PDCD1^hi^ CD200^hi^: logFC > 0.3 and log *p*‐value >2; other: logFC < −0.3 and log *p*‐value >2). The EMT pathway–related genes was highlighted; (B) gene set enrichment analysis (GSEA) showing that the myogenesis pathway was overrepresented in PDCD1^hi^ CD200^hi^ groups; (C) the UMAP plot showing epithelial cells coloured by different tissue; (D) the UMAP plot showing the expression of epithelial cell‐specific genes (EPCAM, KRT18 and KRT8) in epithelial cells; (E) the UMAP plot showing the expression of stem genes (CD44, CD55, KLF4 and ALDH1A1) in epithelial cells; (F) the PAGA (partition‐based graph abstraction) algorithm showing the differentiation trajectories of three epithelial cell subclusters; (G) the heat map showing the evolutionary relationship among the three epithelial cell subclusters.

Supporting Information

**Figure S6 The expression of GAS6 in the PD1hi CD200low CD4^+^ exhausted T cells**. Multiplex immunofluorescence staining was performed for DAPI (blue), CD4 (white), PD1 (green), CD200 (red) and GAS6 (lawngreen).

Supporting Information

**Figure S7 The GAS6–AXL axis predicted the poor survival rates in bladder cancer: (A)** disease‐free survival and progression‐free interval between the high and low expressions of GAS6 groups in TCGA‐BLCA; (B) disease‐free survival and progression‐free interval between the high and low expressions of AXL groups in TCGA‐BLCA; (C) based on the TCGA data, the expression levels of the GAS6 gene in the PDCD1^low^, PDCD1^hi^ CD200^low^ and PDCD1^hi^ CD200^hi^ groups. **** *p*‐Value ≤0.0001; (D) the expression levels of the GAS6 gene among the different groups of the N pathological stages (stage N) of TCGA‐BLCA. ***p‐Value ≤0.001; (E) correlation of GAS6 expression and metastatic potential in cell lines of BLCA; (F) the EMT pathway obtained by enriching the hallmark pathway differentially expressed genes between GAS6^hi^ and GAS6^low^ groups in cell lines of BLCA.

Supporting Information

Supporting Information

Supporting Information

Supporting Information

Supporting Information

Supporting Information

Supporting Information

Supporting Information

## Data Availability

The data supporting the findings of this study are accessible within the article and its supplementary materials.

## References

[1] Siegel R , Miller K , Fuchs H , Jemal A . Cancer statistics, 2022. CA: Cancer J Clin. 2022;72(1):7‐33. 35020204 10.3322/caac.21708

[2] Sung H , Ferlay J , Siegel RL , et al. Global cancer statistics 2020: GLOBOCAN estimates of incidence and mortality worldwide for 36 cancers in 185 countries. CA Cancer J Clin. 2021;71(3):209‐249. 33538338 10.3322/caac.21660

[3] Afonso J , Santos L , Longatto‐Filho A , Baltazar F . Competitive glucose metabolism as a target to boost bladder cancer immunotherapy. Nat Rev Urol. 2020;17(2):77‐106. 31953517 10.1038/s41585-019-0263-6

[4] Hodi F , O'Day S , McDermott D , et al. Improved survival with ipilimumab in patients with metastatic melanoma. N Engl J Med. 2010;363(8):711‐723. 20525992 10.1056/NEJMoa1003466 PMC3549297

[5] Herbst R , Soria J , Kowanetz M , et al. Predictive correlates of response to the anti‐PD‐L1 antibody MPDL3280A in cancer patients. Nature. 2014;515(7528):563‐567. 25428504 10.1038/nature14011 PMC4836193

[6] Powles T , Eder J , Fine G , et al. MPDL3280A (anti‐PD‐L1) treatment leads to clinical activity in metastatic bladder cancer. Nature. 2014;515(7528):558‐562. 25428503 10.1038/nature13904

[7] Waldman A , Fritz J , Lenardo M . A guide to cancer immunotherapy: from T cell basic science to clinical practice. Nat Rev Immunol. 2020;20(11):651‐668. 32433532 10.1038/s41577-020-0306-5 PMC7238960

[8] Hargadon K , Johnson C , Williams C . Immune checkpoint blockade therapy for cancer: an overview of FDA‐approved immune checkpoint inhibitors. Int Immunopharmacol. 2018;62:29‐39. 29990692 10.1016/j.intimp.2018.06.001

[9] Mariathasan S , Turley S , Nickles D , et al. TGFβ attenuates tumour response to PD‐L1 blockade by contributing to exclusion of T cells. Nature. 2018;554(7693):544‐548. 29443960 10.1038/nature25501 PMC6028240

[10] Oh D , Kwek S , Raju S , et al. Intratumoral CD4 T cells mediate anti‐tumor cytotoxicity in human bladder cancer. Cell. 2020;181(7):1612‐1625.e13. 32497499 10.1016/j.cell.2020.05.017 PMC7321885

[11] Balança C , Salvioni A , Scarlata C , et al. PD‐1 blockade restores helper activity of tumor‐infiltrating, exhausted PD‐1hiCD39+ CD4 T cells. JCI Insight. 2021;6(2):e142513. 33332284 10.1172/jci.insight.142513 PMC7934837

[12] Oh D , Fong L . Cytotoxic CD4 T cells in cancer: expanding the immune effector toolbox. Immunity. 2021;54(12):2701‐2711. 34910940 10.1016/j.immuni.2021.11.015 PMC8809482

[13] Zhu J , Yamane H , Paul W . Differentiation of effector CD4 T cell populations (*). Annu Rev Immunol. 2010;28:445‐489. 20192806 10.1146/annurev-immunol-030409-101212 PMC3502616

[14] Tian L , Goldstein A , Wang H , et al. Mutual regulation of tumour vessel normalization and immunostimulatory reprogramming. Nature. 2017;544(7649):250‐254. 28371798 10.1038/nature21724 PMC5788037

[15] Schiavoni G , Gabriele L , Mattei F . The tumor microenvironment: a pitch for multiple players. Front Oncol. 2013;3:90. 23616948 10.3389/fonc.2013.00090 PMC3628362

[16] Goebel L , Grage‐Griebenow E , Gorys A , et al. CD4 T cells potently induce epithelial‐mesenchymal‐transition in premalignant and malignant pancreatic ductal epithelial cells‐novel implications of CD4 T cells in pancreatic cancer development. Oncoimmunology. 2015;4(4):e1000083. 26137395 10.1080/2162402X.2014.1000083 PMC4485733

[17] Burr S , Dazzi F , Garden O . Mesenchymal stromal cells and regulatory T cells: the Yin and Yang of peripheral tolerance? Immunol Cell Biol. 2013;91(1):12‐18. 23146942 10.1038/icb.2012.60

[18] Chaudhary B , Elkord E . Regulatory T cells in the tumor microenvironment and cancer progression: role and therapeutic targeting. Vaccines. 2016;4(3):28. 27509527 10.3390/vaccines4030028 PMC5041022

[19] Caza T , Landas S . Functional and phenotypic plasticity of CD4(+) T cell subsets. BioMed Res Int. 2015;2015:521957. 26583116 10.1155/2015/521957 PMC4637038

[20] Butler A , Hoffman P , Smibert P , Papalexi E , Satija R . Integrating single‐cell transcriptomic data across different conditions, technologies, and species. Nat Biotechnol. 2018;36(5):411‐420. 29608179 10.1038/nbt.4096 PMC6700744

[21] McGinnis C , Murrow L , Gartner Z . DoubletFinder: doublet detection in single‐cell RNA sequencing data using artificial nearest neighbors. Cell Syst. 2019;8(4):329‐337.e4. 30954475 10.1016/j.cels.2019.03.003 PMC6853612

[22] Korsunsky I , Millard N , Fan J , et al. Fast, sensitive and accurate integration of single‐cell data with Harmony. Nat Methods. 2019;16(12):1289‐1296. 31740819 10.1038/s41592-019-0619-0 PMC6884693

[23] Rose T , Weir W , Mayhew G , et al. Fibroblast growth factor receptor 3 alterations and response to immune checkpoint inhibition in metastatic urothelial cancer: a real world experience. Br J Cancer. 2021;125(9):1251‐1260. 34294892 10.1038/s41416-021-01488-6 PMC8548561

[24] Riaz N , Havel J , Makarov V , et al. Tumor and microenvironment evolution during immunotherapy with nivolumab. Cell. 2017;171(4):934‐949.e16. 29033130 10.1016/j.cell.2017.09.028 PMC5685550

[25] Jung H , Kim H , Kim J , et al. DNA methylation loss promotes immune evasion of tumours with high mutation and copy number load. Nat Commun. 2019;10(1):4278. 31537801 10.1038/s41467-019-12159-9 PMC6753140

[26] Ritchie M , Phipson B , Wu D , et al. Limma powers differential expression analyses for RNA‐sequencing and microarray studies. Nucleic Acids Res. 2015;43(7):e47. 25605792 10.1093/nar/gkv007 PMC4402510

[27] Yu G , Wang L , Han Y , He Q . clusterProfiler: an R package for comparing biological themes among gene clusters. OMICS: J Integr Biol. 2012;16(5):284‐287. 10.1089/omi.2011.0118 PMC3339379 22455463

[28] Aran D , Hu Z , Butte A . xCell: digitally portraying the tissue cellular heterogeneity landscape. Genome Biol. 2017;18(1):220. 29141660 10.1186/s13059-017-1349-1 PMC5688663

[29] Jin S , Guerrero‐Juarez C , Zhang L , et al. Inference and analysis of cell‐cell communication using CellChat. Nat Commun. 2021;12(1):1088. 33597522 10.1038/s41467-021-21246-9 PMC7889871

[30] Bergen V , Lange M , Peidli S , Wolf F , Theis F . Generalizing RNA velocity to transient cell states through dynamical modeling. Nat Biotechnol. 2020;38(12):1408‐1414. 32747759 10.1038/s41587-020-0591-3

[31] Wolf F , Hamey F , Plass M , et al. PAGA: graph abstraction reconciles clustering with trajectory inference through a topology preserving map of single cells. Genome Biol. 2019;20(1):59. 30890159 10.1186/s13059-019-1663-x PMC6425583

[32] Qiu X , Mao Q , Tang Y , et al. Reversed graph embedding resolves complex single‐cell trajectories. Nat Methods. 2017;14(10):979‐982. 28825705 10.1038/nmeth.4402 PMC5764547

[33] Wu J , Wu Y , Guo Q , et al. SPOP promotes cervical cancer progression by inducing the movement of PD‐1 away from PD‐L1 in spatial localization. J Transl Med. 2022;20(1):384. 36042498 10.1186/s12967-022-03574-6 PMC9429754

[34] Liu Y , He S , Wang X , et al. Tumour heterogeneity and intercellular networks of nasopharyngeal carcinoma at single cell resolution. Nat Commun. 2021;12(1):741. 33531485 10.1038/s41467-021-21043-4 PMC7854640

[35] Bai Y , Chen D , Cheng C , et al. Immunosuppressive landscape in hepatocellular carcinoma revealed by single‐cell sequencing. Front Immunol. 2022;13:950536. 35967424 10.3389/fimmu.2022.950536 PMC9365996

[36] Bassez A , Vos H , Van Dyck L , et al. A single‐cell map of intratumoral changes during anti‐PD1 treatment of patients with breast cancer. Nat Med. 2021;27(5):820‐832. 33958794 10.1038/s41591-021-01323-8

[37] Iwakura Y , Ishigame H , Saijo S , Nakae S . Functional specialization of interleukin‐17 family members. Immunity. 2011;34(2):149‐162. 21349428 10.1016/j.immuni.2011.02.012

[38] Lluis A , Ballenberger N , Illi S , et al. Regulation of TH17 markers early in life through maternal farm exposure. The J Allergy Clin Immunol. 2014;133(3):864‐871. 24275363 10.1016/j.jaci.2013.09.030

[39] Vinuesa C , Linterman M , Yu D , MacLennan I . Follicular helper T cells. Annu Rev Immunol. 2016;34:335‐368. 26907215 10.1146/annurev-immunol-041015-055605

[40] Choi Y , Gullicksrud J , Xing S , et al. LEF‐1 and TCF‐1 orchestrate T(FH) differentiation by regulating differentiation circuits upstream of the transcriptional repressor Bcl6. Nat Immunol. 2015;16(9):980‐990. 26214741 10.1038/ni.3226 PMC4545301

[41] Jogdand G , Mohanty S , Devadas S . Regulators of Tfh cell differentiation. Front Immunol. 2016;7:520. 27933060 10.3389/fimmu.2016.00520 PMC5120123

[42] He J , Xiong X , Yang H , et al. Defined tumor antigen‐specific T cells potentiate personalized TCR‐T cell therapy and prediction of immunotherapy response. Cell Res. 2022;32(6):530‐542. 35165422 10.1038/s41422-022-00627-9 PMC9160085

[43] Sen D , Kaminski J , Barnitz R , et al. The epigenetic landscape of T cell exhaustion. Science (New York, NY). 2016;354(6316):1165‐1169. 10.1126/science.aae0491 PMC5497589 27789799

[44] Gordon S . Alternative activation of macrophages. Nat Rev Immunol. 2003;3(1):23‐35. 12511873 10.1038/nri978

[45] Trinchieri G . Interleukin‐12 and the regulation of innate resistance and adaptive immunity. Nat Rev Immunol. 2003;3(2):133‐146. 12563297 10.1038/nri1001

[46] Rahma O , Hodi F . The intersection between tumor angiogenesis and immune suppression. Clin Cancer Res. 2019;25(18):5449‐5457. 30944124 10.1158/1078-0432.CCR-18-1543

[47] Ferrara N . Vascular endothelial growth factor: basic science and clinical progress. Endocr Rev. 2004;25(4):581‐611. 15294883 10.1210/er.2003-0027

[48] Apte R , Chen D , Ferrara N . VEGF in signaling and disease: beyond discovery and development. Cell. 2019;176(6):1248‐1264. 30849371 10.1016/j.cell.2019.01.021 PMC6410740

[49] Togashi K , Shin Y , Imamura Y . Non‐triple helical form of type IV collagen alpha1 chain suppresses vascular endothelial‐cadherin mediated cell‐to‐cell junctions. J Biochem. 2022;172(3):165‐175. 35687058 10.1093/jb/mvac050

[50] Pradella D , Naro C , Sette C , Ghigna C . EMT and stemness: flexible processes tuned by alternative splicing in development and cancer progression. Mol Cancer. 2017;16(1):8. 28137272 10.1186/s12943-016-0579-2 PMC5282733

[51] Zhao B , Roundtree I , He C . Post‐transcriptional gene regulation by mRNA modifications. Nat Rev Mol Cell Biol. 2017;18(1):31‐42. 27808276 10.1038/nrm.2016.132 PMC5167638

[52] Liu J , Harada B , He C . Regulation of gene expression by N‐methyladenosine in cancer. Trends Cell Biol. 2019;29(6):487‐499. 30940398 10.1016/j.tcb.2019.02.008 PMC6527461

[53] Zhou Y , Zeng P , Li Y , Zhang Z , Cui Q . SRAMP: prediction of mammalian N6‐methyladenosine (m6A) sites based on sequence‐derived features. Nucleic Acids Res. 2016;44(10):e91. 26896799 10.1093/nar/gkw104 PMC4889921

[54] Oliveira G , Stromhaug K , Cieri N , et al. Landscape of helper and regulatory antitumour CD4 T cells in melanoma. Nature. 2022;605(7910):532‐538. 35508657 10.1038/s41586-022-04682-5 PMC9815755

[55] Guo X , Zhang Y , Zheng L , et al. Global characterization of T cells in non‐small‐cell lung cancer by single‐cell sequencing. Nat Med. 2018;24(7):978‐985. 29942094 10.1038/s41591-018-0045-3

[56] Zheng C , Zheng L , Yoo J , et al. Landscape of infiltrating T cells in liver cancer revealed by single‐cell sequencing. Cell. 2017;169(7):1342‐1356.e16. 28622514 10.1016/j.cell.2017.05.035

[57] Zheng L , Qin S , Si W , et al. Pan‐cancer single‐cell landscape of tumor‐infiltrating T cells. Science (New York, NY). 2021;374(6574):abe6474. 10.1126/science.abe6474 34914499

[58] Hoek R , Ruuls S , Murphy C , et al. Down‐regulation of the macrophage lineage through interaction with OX2 (CD200). Science (New York, NY). 2000;290(5497):1768‐1771. 10.1126/science.290.5497.1768 11099416

[59] Wright G , Jones M , Puklavec M , Brown M , Barclay A . The unusual distribution of the neuronal/lymphoid cell surface CD200 (OX2) glycoprotein is conserved in humans. Immunology. 2001;102(2):173‐179. 11260322 10.1046/j.1365-2567.2001.01163.x PMC1783166

[60] Bukovský A , Presl J , Zidovský J . Association of some cell surface antigens of lymphoid cells and cell surface differentiation antigens with early rat pregnancy. Immunology. 1984;52(4):631‐640. 6146566 PMC1454680

[61] Chamera K , Szuster‐Głuszczak M , Trojan E , Basta‐Kaim A . Maternal immune activation sensitizes male offspring rats to lipopolysaccharide‐induced microglial deficits involving the dysfunction of CD200‐CD200R and CX3CL1‐CX3CR1 systems. Cells. 2020;9(7):1676. 32664639 10.3390/cells9071676 PMC7407118

[62] Liu J , Hu A , Zhu J , Yu J , Talebian F , Bai X . CD200‐CD200R pathway in the regulation of tumor immune microenvironment and immunotherapy. Adv Exp Med Biol. 2020;1223:155‐165. 32030689 10.1007/978-3-030-35582-1_8 PMC7339106

[63] Rygiel T , Karnam G , Goverse G , et al. CD200‐CD200R signaling suppresses anti‐tumor responses independently of CD200 expression on the tumor. Oncogene. 2012;31(24):2979‐2988. 22020332 10.1038/onc.2011.477

[64] Ren Y , Yang B , Yin Y , et al. Aberrant CD200/CD200R1 expression and its potential role in Th17 cell differentiation, chemotaxis and osteoclastogenesis in rheumatoid arthritis. Rheumatology (Oxford, England). 2015;54(4):712‐721. 25261692 10.1093/rheumatology/keu362

[65] Ustun C , Miller J , Munn D , Weisdorf D , Blazar B . Regulatory T cells in acute myelogenous leukemia: is it time for immunomodulation? Blood. 2011;118(19):5084‐5095. 21881045 10.1182/blood-2011-07-365817 PMC3217399

[66] Ismail A , Donia H , Ghatesh H , Farid C . CD200/CD200 receptor axis in psoriasis vulgaris. PLoS One. 2020;15(3):e0230621. 32203537 10.1371/journal.pone.0230621 PMC7089552

[67] Dong C . Cytokine regulation and function in T cells. Annu Rev Immunol. 2021;39:51‐76. 33428453 10.1146/annurev-immunol-061020-053702

[68] Togashi Y , Shitara K , Nishikawa H . Regulatory T cells in cancer immunosuppression – implications for anticancer therapy. Nat Rev Clin Oncol. 2019;16(6):356‐371. 30705439 10.1038/s41571-019-0175-7

[69] Tanaka A , Sakaguchi S . Regulatory T cells in cancer immunotherapy. Cell Res. 2017;27(1):109‐118. 27995907 10.1038/cr.2016.151 PMC5223231

[70] Allen E , Jabouille A , Rivera L , et al. Combined antiangiogenic and anti‐PD‐L1 therapy stimulates tumor immunity through HEV formation. Sci Transl Med. 2017;9(385):eaak9679. 28404866 10.1126/scitranslmed.aak9679 PMC5554432

[71] Qiao S , Hou Y , Rong Q , Han B , Liu P . Tregs are involved in VEGFA/VASH1‐related angiogenesis pathway in ovarian cancer. Transl Oncol. 2023;32:101665. 37018867 10.1016/j.tranon.2023.101665 PMC10106963

[72] Lužnik Z , Anchouche S , Dana R , Yin J . Regulatory T cells in angiogenesis. J Immunol (Baltimore, MD: 1950). 2020;205(10):2557‐2565. 10.4049/jimmunol.2000574 PMC7664842 33168598

[73] Facciabene A , Motz G , Coukos G . T‐regulatory cells: key players in tumor immune escape and angiogenesis. Cancer Res. 2012;72(9):2162‐2171. 22549946 10.1158/0008-5472.CAN-11-3687 PMC3342842

[74] Wang X , Zhou W , Luo X , Tao Y , Li D . Synergistic effect of regulatory T cells and proinflammatory cytokines in angiogenesis in the endometriotic milieu. Hum Reprod (Oxford, UK). 2017;32(6):1304‐1317. 10.1093/humrep/dex067 28383711

[75] Mu Q , Najafi M . Modulation of the tumor microenvironment (TME) by melatonin. Eur J Pharmacol. 2021;907:174365. 34302814 10.1016/j.ejphar.2021.174365

[76] Xu Y , Dong X , Qi P , et al. Sox2 communicates with tregs through CCL1 to promote the stemness property of breast cancer cells. Stem Cells (Dayton, OH). 2017;35(12):2351‐2365. 10.1002/stem.2720 PMC5958902 29044882

[77] Huang Y , Wang F , Wang T , et al. Tumor‐infiltrating FoxP3+ Tregs and CD8+ T cells affect the prognosis of hepatocellular carcinoma patients. Digestion. 2012;86(4):329‐337. 23207161 10.1159/000342801

[78] Facciabene A , Peng X , Hagemann I , et al. Tumour hypoxia promotes tolerance and angiogenesis via CCL28 and T(reg) cells. Nature. 2011;475(7355):226‐230. 21753853 10.1038/nature10169

[79] Leung O , Li J , Li X , et al. Regulatory T cells promote apelin‐mediated sprouting angiogenesis in type 2 diabetes. Cell Rep. 2018;24(6):1610‐1626. 30089270 10.1016/j.celrep.2018.07.019

[80] D'Alessio F , Zhong Q , Jenkins J , Moldobaeva A , Wagner E . Lung angiogenesis requires CD4(+) forkhead homeobox protein‐3(+) regulatory T cells. Am J Respir Cell Mol Biol. 2015;52(5):603‐610. 25275926 10.1165/rcmb.2014-0278OC PMC4491140

[81] Carmeliet P , De Smet F , Loges S , Mazzone M . Branching morphogenesis and antiangiogenesis candidates: tip cells lead the way. Nat Rev Clin Oncol. 2009;6(6):315‐326. 19483738 10.1038/nrclinonc.2009.64

[82] De Bock K , Georgiadou M , Carmeliet P . Role of endothelial cell metabolism in vessel sprouting. Cell Metab. 2013;18(5):634‐647. 23973331 10.1016/j.cmet.2013.08.001

[83] Eelen G , Cruys B , Welti J , De Bock K , Carmeliet P . Control of vessel sprouting by genetic and metabolic determinants. Trends Endocrinol Metab: TEM. 2013;24(12):589‐596. 24075830 10.1016/j.tem.2013.08.006

[84] Giuliani A , Sarti A , Di Virgilio F . Extracellular nucleotides and nucleosides as signalling molecules. Immunol Lett. 2019;205:16‐24. 30439478 10.1016/j.imlet.2018.11.006

[85] Dosch M , Gerber J , Jebbawi F , Beldi G . Mechanisms of ATP release by inflammatory cells. Int J Mol Sci. 2018;19(4):1222. 29669994 10.3390/ijms19041222 PMC5979498

[86] Gendaszewska‐Darmach E , Szustak M . Thymidine 5′‐O‐monophosphorothioate induces HeLa cell migration by activation of the P2Y6 receptor. Purinergic Signal. 2016;12(2):199‐209. 26746211 10.1007/s11302-015-9492-1 PMC4854834

[87] Nishimura A , Sunggip C , Tozaki‐Saitoh H , et al. Purinergic P2Y6 receptors heterodimerize with angiotensin AT1 receptors to promote angiotensin II‐induced hypertension. Sci Signal. 2016;9(411):ra7. 26787451 10.1126/scisignal.aac9187

[88] Campwala H , Sexton D , Crossman D , Fountain S . P2Y₆ receptor inhibition perturbs CCL2‐evoked signalling in human monocytic and peripheral blood mononuclear cells. J Cell Sci. 2014;127:4964‐4973. 25271060 10.1242/jcs.159012 PMC4231309

[89] Dongre A , Weinberg R . New insights into the mechanisms of epithelial‐mesenchymal transition and implications for cancer. Nat Rev Mol Cell Biol. 2019;20(2):69‐84. 30459476 10.1038/s41580-018-0080-4

[90] Xiong S , Pan X , Xu L , et al. Regulatory T cells promote β‐catenin–mediated epithelium‐to‐mesenchyme transition during radiation‐induced pulmonary fibrosis. Int J Radiat Oncol Biol Phys. 2015;93(2):425‐435. 26253394 10.1016/j.ijrobp.2015.05.043

[91] Shi C , Chen Y , Chen Y , Yang Y , Bing W , Qi J . CD4 CD25 regulatory T cells promote hepatocellular carcinoma invasion via TGF‐β1‐induced epithelial‐mesenchymal transition. Onco Targets Ther. 2019;12:279‐289. 30643426 10.2147/OTT.S172417 PMC6314313

[92] Oh E , Hong J , Yun C . Regulatory T cells induce metastasis by increasing Tgf‐β and enhancing the epithelial–mesenchymal transition. Cells. 2019;8(11):1387. 31690033 10.3390/cells8111387 PMC6912455

[93] Tanaka M , Siemann D . Gas6/Axl signaling pathway in the tumor immune microenvironment. Cancers. 2020;12(7):1850. 32660000 10.3390/cancers12071850 PMC7408754

[94] Graham D , DeRyckere D , Davies K , Earp H . The TAM family: phosphatidylserine sensing receptor tyrosine kinases gone awry in cancer. Nat Rev Cancer. 2014;14(12):769‐785. 25568918 10.1038/nrc3847

[95] Wilson C , Ye X , Pham T , et al. AXL inhibition sensitizes mesenchymal cancer cells to antimitotic drugs. Cancer Res. 2014;74(20):5878‐5890. 25125659 10.1158/0008-5472.CAN-14-1009

[96] Zhao G , Zheng J , Bian J , et al. Growth arrest‐specific 6 enhances the suppressive function of CD4CD25 regulatory T cells mainly through Axl receptor. Mediators Inflamm. 2017;2017:6848430. 28270700 10.1155/2017/6848430 PMC5320320

[97] Fridell Y , Villa J , Attar E , Liu E . GAS6 induces Axl‐mediated chemotaxis of vascular smooth muscle cells. J Biol Chem. 1998;273(12):7123‐7126. 9507025 10.1074/jbc.273.12.7123

[98] Zaccara S , Ries R , Jaffrey S . Reading, writing and erasing mRNA methylation. Nat Rev Mol Cell Biol. 2019;20(10):608‐624. 31520073 10.1038/s41580-019-0168-5

[99] Jin J , Xu C , Wu S , et al. GAS6mA demethylase ALKBH5 restrains PEDV infection by regulating expression in porcine alveolar macrophages. Int J Mol Sci. 2022;23(11):6191. 35682869 10.3390/ijms23116191 PMC9181496

[100] Bao X , Zhang Y , Li H , et al. RM2Target: a comprehensive database for targets of writers, erasers and readers of RNA modifications. Nucleic Acids Res. 2022;51:D269–D279. 10.1093/nar/gkac945 PMC9825529 36300630

