# Supplementary figures and images for: PD1hi CD200hi CD4+ exhausted T cell increase immunotherapy resistance and tumour progression by promoting epithelial–mesenchymal transition in bladder cancer
Source: Clin Transl Med. 2023 Jun 14;13(6):e1303. doi: 10.1002/ctm2.1303 (PMC10265167; doi:10.1002/ctm2.1303)

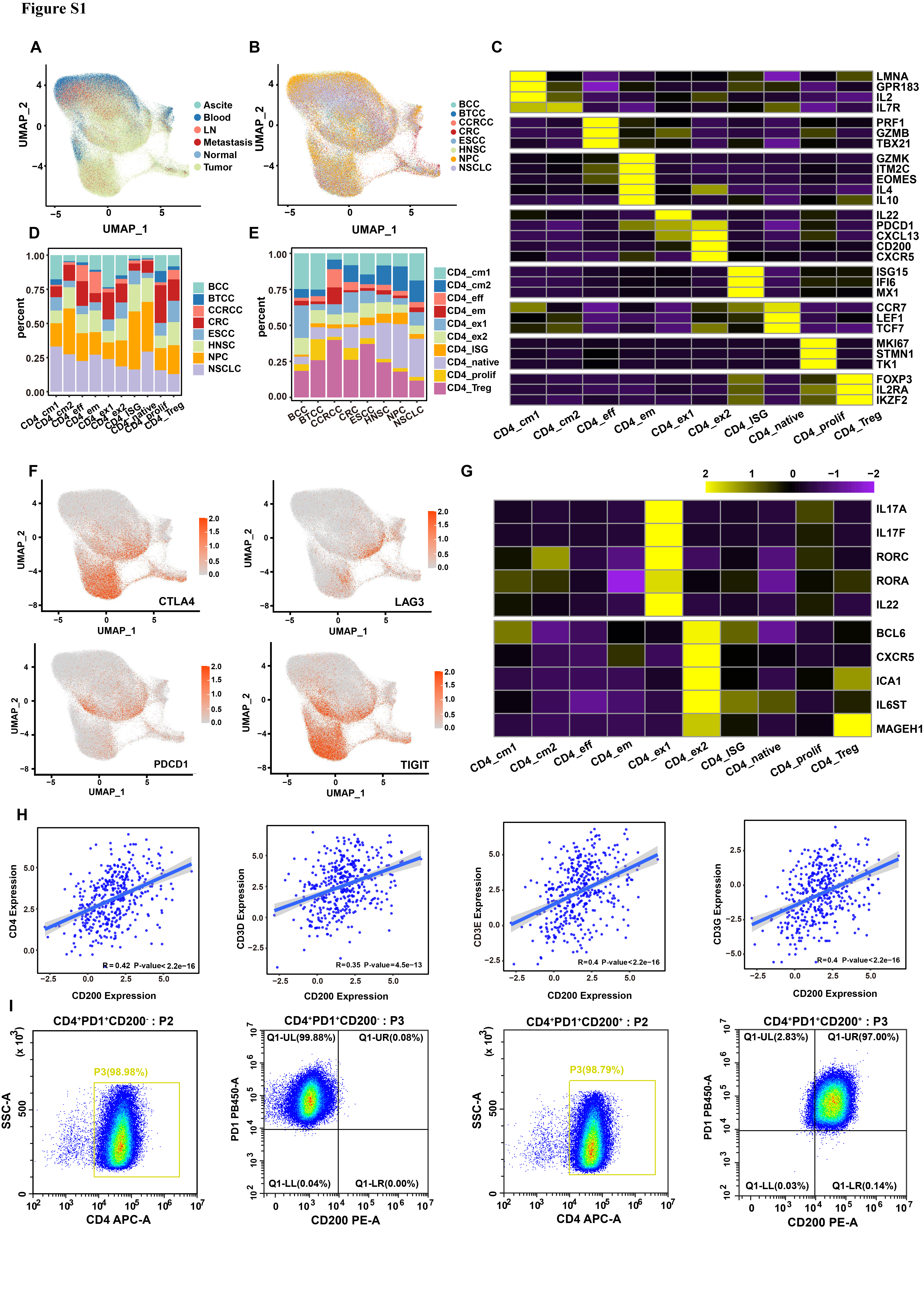

Supplement: Supplementary file 1 — Figure S1 The PD1hi CD200hi CD4+ exhausted T cells in the tumour microenvironment: (A) the UMAP plot showing pan‐CD4+ T cells coloured by tissue types. Each dot indicates a single cell. Colour‐coded for the tissue type; (B) the UMAP plot showing the CD4+ T cells labelling cancer types; (C) heat map showing the expression of signature genes in CD4 subclusters; (D) barplot showing the percentage of cancer types in each cluster of pan‐CD4 cells; (E) barplot showing the percentage of each cluster of pan‐CD4 cells in each dataset; (F) the UMAP plot showing the exhausted‐related genes (PDCD1, CTLA4, LAG3 and TIGIT) expression in pan‐CD4+ T cells atlas; (G) the boxplot showing the expression of Th17‐related and Tfh‐related gene in CD4_Tex1 and CD4_Tex2 clusters; (H) correlation of the expression of CD200 and CD4+ T‐cell signature genes (CD4, CD3D, CD3E and CD3G) in TCGA‐BLCA; (I) validation of PD1hi CD200low CD4+ T cells and PD‐1hi CD200hi CD4+ T cells by flow cytometry. BCC; basal cell carcinoma; BTCC, bladder transitional cell carcinoma; CCRCC, clear cell renal carcinoma; CRC, colorectal cancer; ESCC, oesophageal squamous cell carcinoma; HNSC, head and neck squamous cell carcinoma; Lung, lung cancer, NPC: nasopharyngeal carcinoma. [file CTM2-13-e1303-s020.tif]

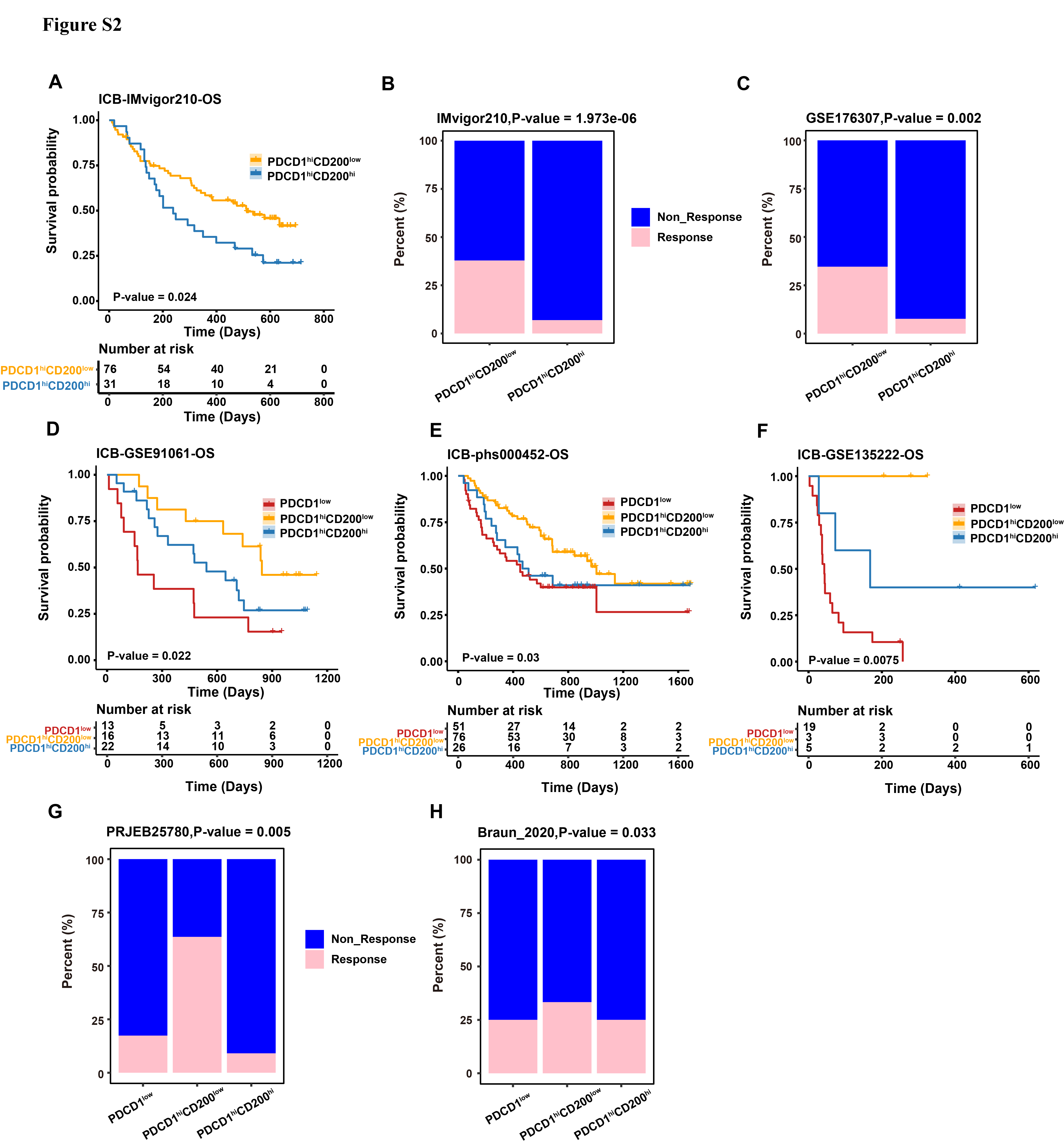

Supplement: Supplementary file 3 — Figure S2 The PD1hi CD200hi CD4+ exhausted T cells predicted the poor response to immunotherapy in pancancer: (A–C) overall survival of patients with immunotherapy in PDCD1low, PDCD1hi CD200low and PDCD1hi CD200hi groups in the GSE91061 and phs00052 (melanoma), and GSE135222 (NSCLC) cohorts; (D and E) boxplots showing the proportion of responders among PDCD1low, PDCD1hi CD200low and PDCD1hi CD200hi groups in the PRJEB25780 (STAD) and Braun_2020 (RCC) cohorts; (F) overall survival of patients with immunotherapy in PDCD1hi CD200low and PDCD1hi CD200hi groups in the IMvigor210 cohorts; (G and H) boxplots showing the proportion of responders between PDCD1hi CD200low and PDCD1hi CD200hi groups in the IMvigor210 and GSE176307 cohorts. [file CTM2-13-e1303-s018.tif]

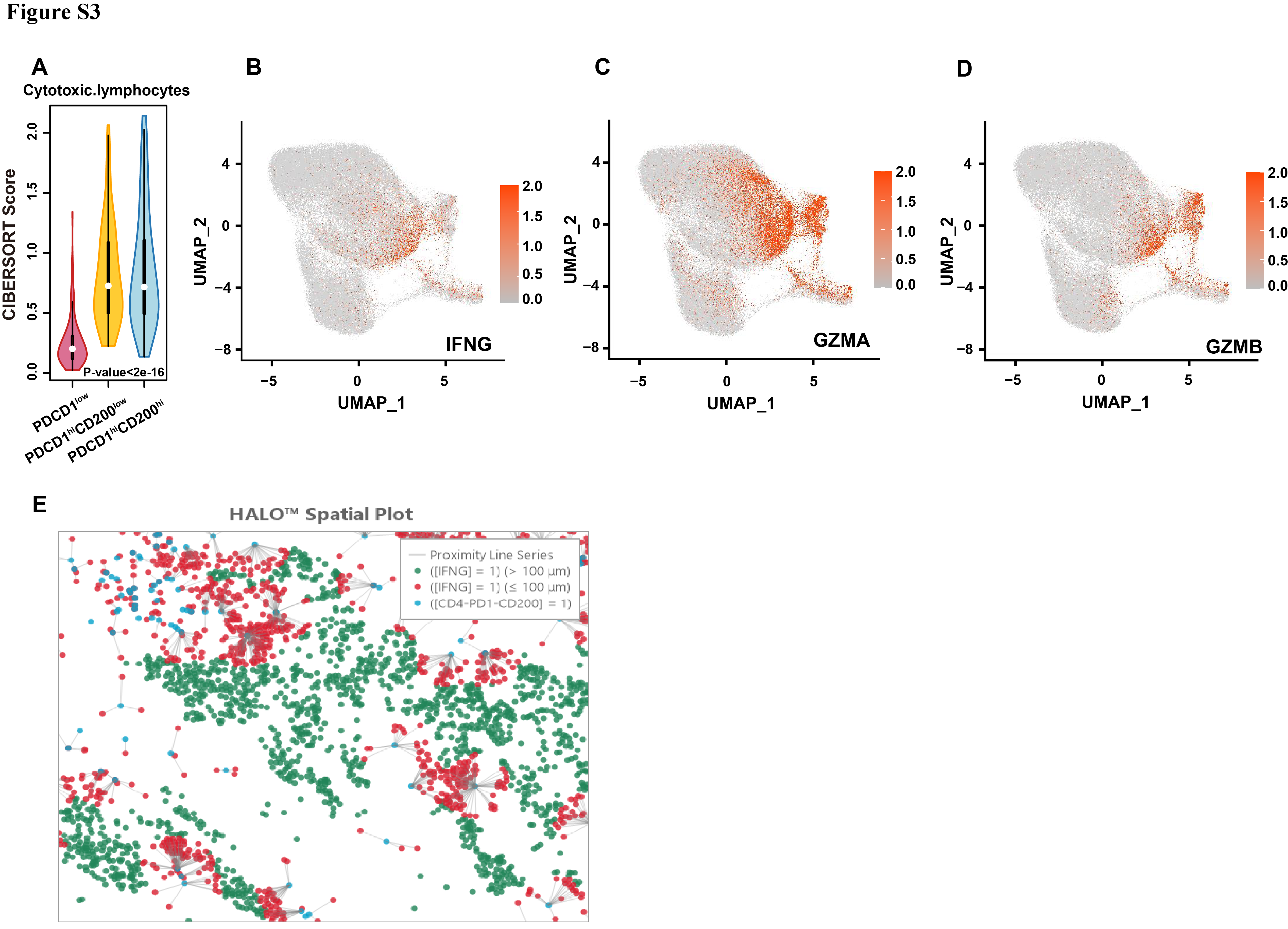

Supplement: Supplementary file 5 — Figure S3 The expression of cytotoxic factors in the PD1hi CD200hi CD4+ exhausted T cells: (A) violin plot showing the estimation of the abundance of cytotoxic lymphocyte in the PDCD1low, PDCD1hi CD200low and PDCD1hi CD200hi groups using CIBERSORT_counter algorithm; (B–D) the UMAP plot showing the expression of genes associated with T‐effector (IFNG, GZMA and GZMB) in pan‐CD4+ T cells; (E) spatial distribution of IFNG+ cells around PD‐1hi CD200hi CD4+ exhausted T cells. [file CTM2-13-e1303-s012.tif]

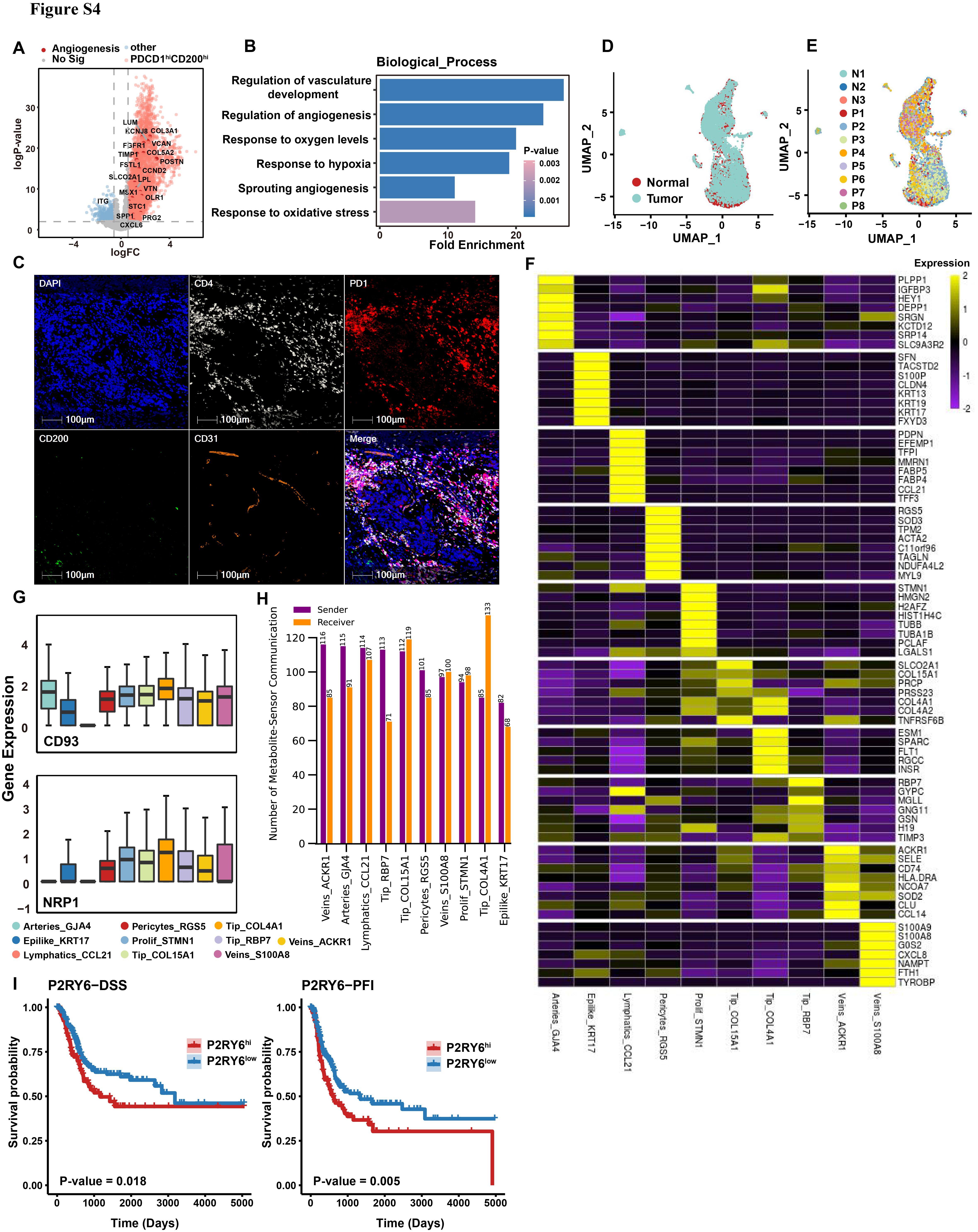

Supplement: Supplementary file 7 — Figure S4 The PD1hi CD200hi CD4+ exhausted T cells induced the angiogenesis in bladder cancer: (A) volcano plot showing differentially expressed genes between PDCD1hi CD200hi and other (PDCD1low and PDCD1hi CD200low) groups (PDCD1hi CD200hi: logFC > 0.3 and log p‐value >2; other: logFC < −0.3 and log p‐value >2). The angiogenesis pathway–related genes were highlighted; (B) barplot showing the pathway enrichment in PDCD1hi CD200hi group in TCGA‐BLCA; (C) multiplex immunofluorescence staining was performed for DAPI (blue), CD4 (white), PD1 (red), CD200 (green) and CD31 (orange); (D) the UMAP plot showing endothelial cells coloured by different tissue; (E) the UMAP plot showing endothelial cells coloured by each patient; (F) the heat map shows the top eight particular genes for each subcluster of endothelial cells; (G) the boxplot showing the angiogenesis‐related genes expressed in subclusters of endothelial cells; (H) barplot showing the number of communications for senders and receivers. The x axis is the subclusters of endothelial cells. The y axis is the number of communications. The orange and purple bars are the number of communications for sender and receiver cells, respectively; (I) disease‐free survival and progression‐free interval between the high and low expressions of P2RY6 groups in TCGA‐BLCA. [file CTM2-13-e1303-s006.tif]

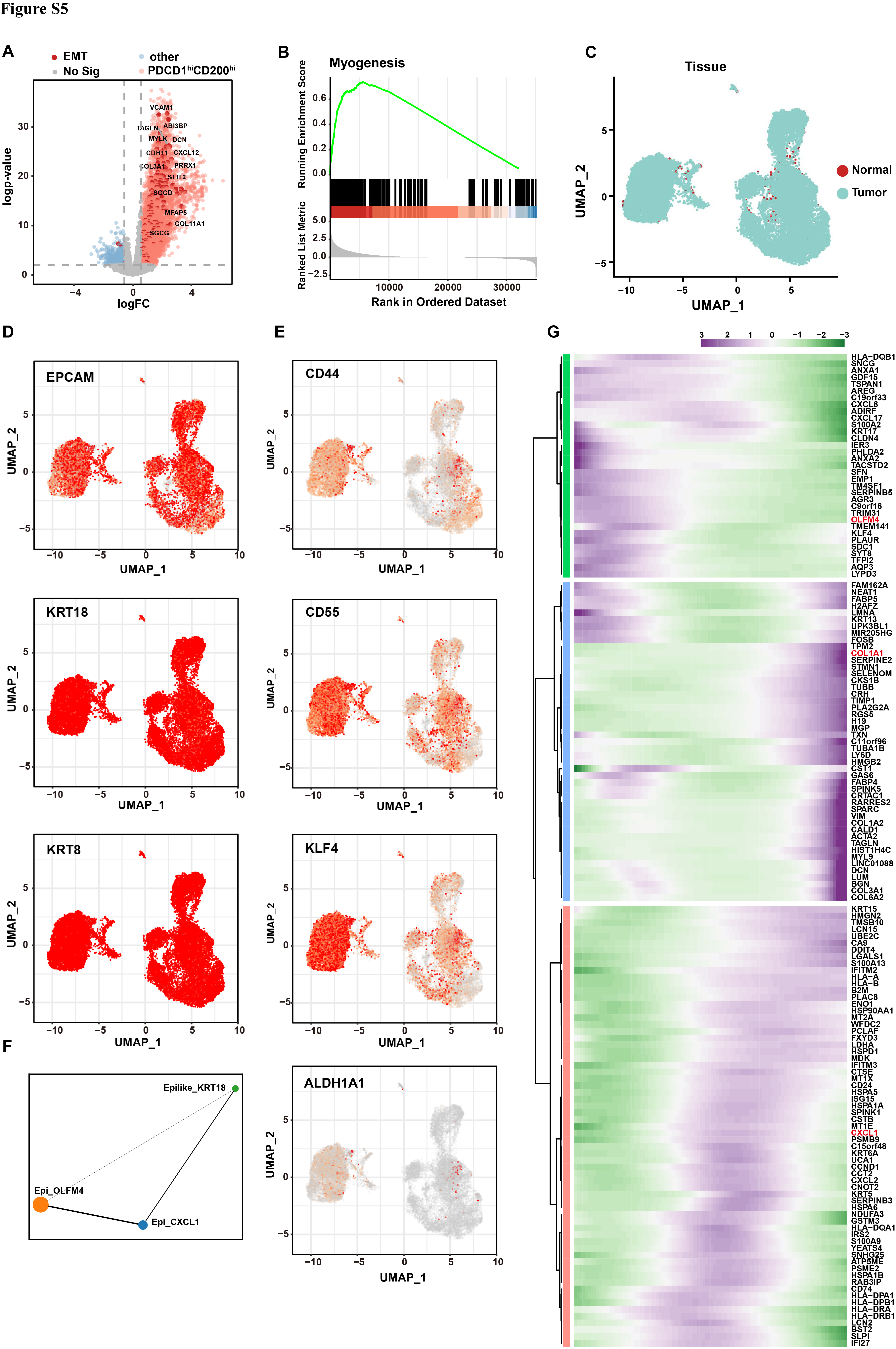

Supplement: Supplementary file 9 — Figure S5 The PD1hi CD200hi CD4+ exhausted T cells promoted the epithelial–mesenchymal transition (EMT) in bladder cancer: (A) volcano map showing differentially expressed genes between PDCD1hi CD200hi and other (PDCD1low and PDCD1hi CD200low) groups (PDCD1hi CD200hi: logFC > 0.3 and log p‐value >2; other: logFC < −0.3 and log p‐value >2). The EMT pathway–related genes was highlighted; (B) gene set enrichment analysis (GSEA) showing that the myogenesis pathway was overrepresented in PDCD1hi CD200hi groups; (C) the UMAP plot showing epithelial cells coloured by different tissue; (D) the UMAP plot showing the expression of epithelial cell‐specific genes (EPCAM, KRT18 and KRT8) in epithelial cells; (E) the UMAP plot showing the expression of stem genes (CD44, CD55, KLF4 and ALDH1A1) in epithelial cells; (F) the PAGA (partition‐based graph abstraction) algorithm showing the differentiation trajectories of three epithelial cell subclusters; (G) the heat map showing the evolutionary relationship among the three epithelial cell subclusters. [file CTM2-13-e1303-s013.tif]

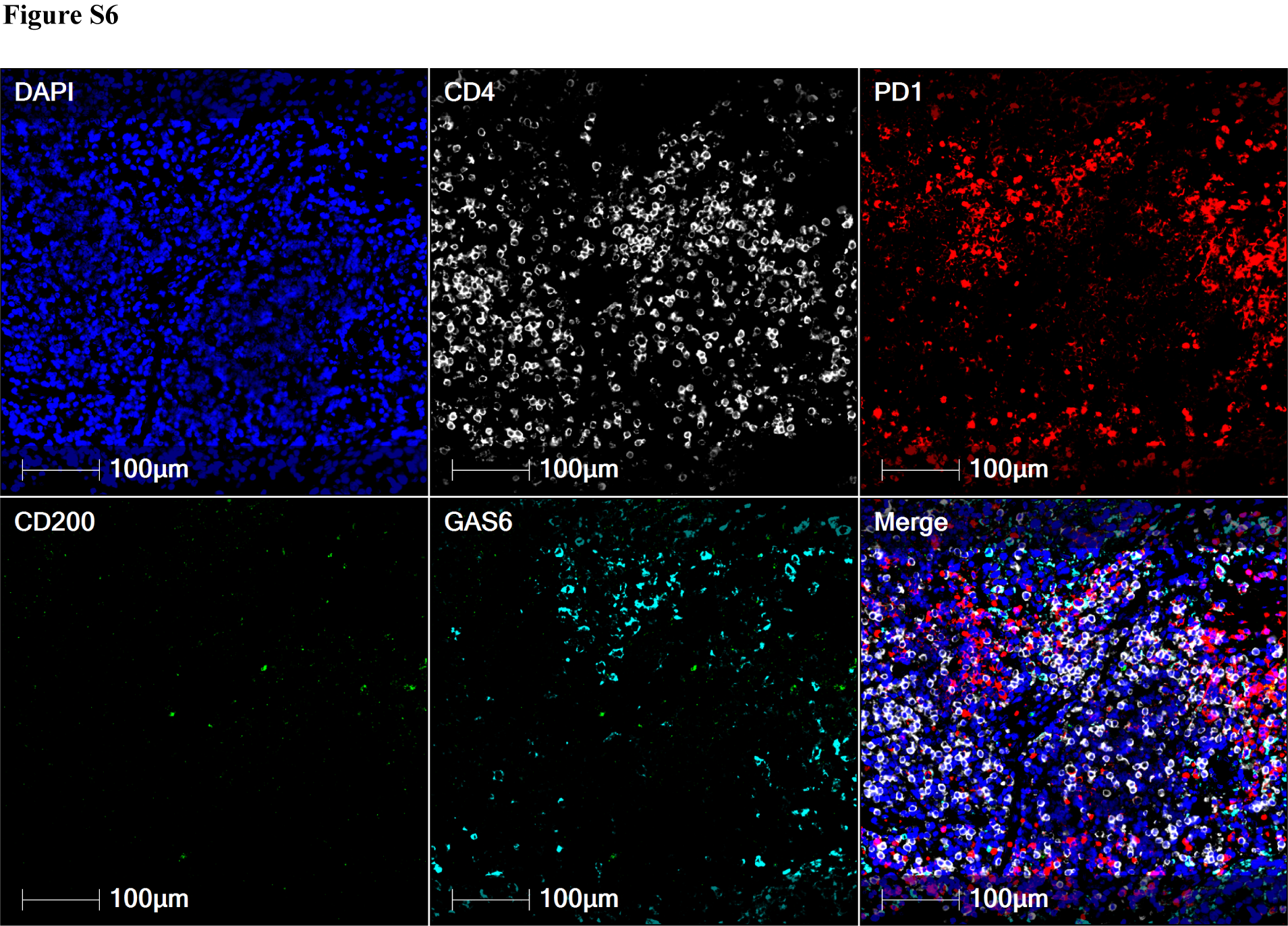

Supplement: Supplementary file 11 — Figure S6 The expression of GAS6 in the PD1hi CD200low CD4+ exhausted T cells. Multiplex immunofluorescence staining was performed for DAPI (blue), CD4 (white), PD1 (green), CD200 (red) and GAS6 (lawngreen). [file CTM2-13-e1303-s003.tif]

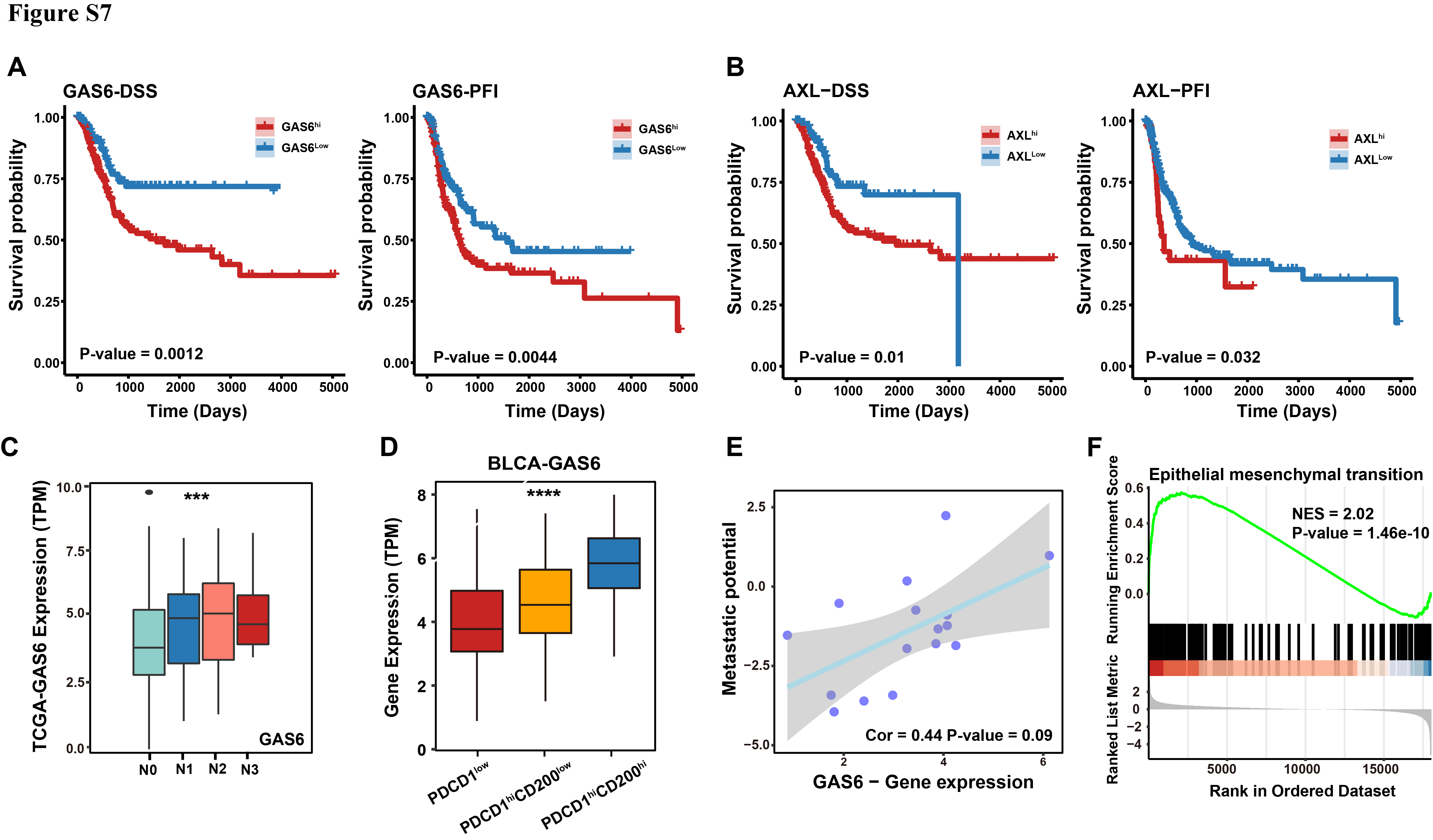

Supplement: Supplementary file 13 — Figure S7 The GAS6–AXL axis predicted the poor survival rates in bladder cancer: (A) disease‐free survival and progression‐free interval between the high and low expressions of GAS6 groups in TCGA‐BLCA; (B) disease‐free survival and progression‐free interval between the high and low expressions of AXL groups in TCGA‐BLCA; (C) based on the TCGA data, the expression levels of the GAS6 gene in the PDCD1low, PDCD1hi CD200low and PDCD1hi CD200hi groups. **** p‐Value ≤0.0001; (D) the expression levels of the GAS6 gene among the different groups of the N pathological stages (stage N) of TCGA‐BLCA. ***p‐Value ≤0.001; (E) correlation of GAS6 expression and metastatic potential in cell lines of BLCA; (F) the EMT pathway obtained by enriching the hallmark pathway differentially expressed genes between GAS6hi and GAS6low groups in cell lines of BLCA. [file CTM2-13-e1303-s004.tif]
